# An Updated Review and Meta Analysis of Lipoprotein Glomerulopathy

**DOI:** 10.3389/fmed.2022.905007

**Published:** 2022-05-06

**Authors:** Meng-shi Li, Yang Li, Yang Liu, Xu-jie Zhou, Hong Zhang

**Affiliations:** ^1^Renal Division, Peking University First Hospital, Beijing, China; ^2^Kidney Genetics Center, Peking University Institute of Nephrology, Beijing, China; ^3^Key Laboratory of Renal Disease, Ministry of Health of China, Beijing, China; ^4^Key Laboratory of Chronic Kidney Disease Prevention and Treatment (Peking University), Ministry of Education, Beijing, China

**Keywords:** lipoprotein glomerulopathy, apolipoprotein E, epidemiology, pathogenesis, meta-analysis, treatment

## Abstract

More than 200 cases of lipoprotein glomerulopathy (LPG) have been reported since it was first discovered 30 years ago. Although relatively rare, LPG is clinically an important cause of nephrotic syndrome and end-stage renal disease. Mutations in the *APOE* gene are the leading cause of LPG. *APOE* mutations are an important determinant of lipid profiles and cardiovascular health in the population and can precipitate dysbetalipoproteinemia and glomerulopathy. Apolipoprotein E-related glomerular disorders include *APOE*2 homozygote glomerulopathy and LPG with heterozygous *APOE* mutations. In recent years, there has been a rapid increase in the number of LPG case reports and some progress in research into the mechanism and animal models of LPG. We consequently need to update recent epidemiological studies and the molecular mechanisms of LPG. This endeavor may help us not only to diagnose and treat LPG in a more personized manner but also to better understand the potential relationship between lipids and the kidney.

## Introduction

**Figure 1:**
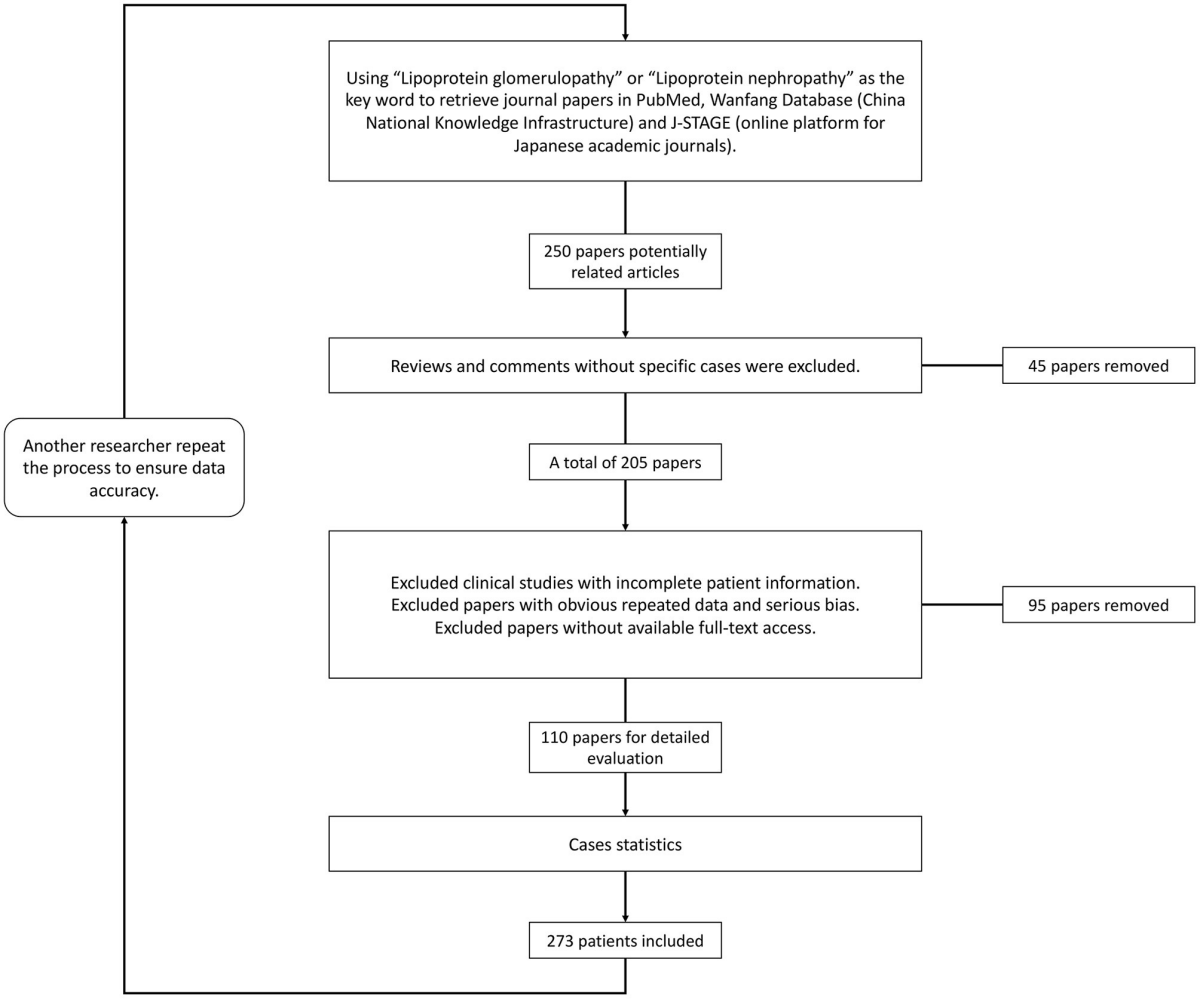
Flowchart of the literature review and case screening.

Since it was first described in 1989 (1), lipoprotein glomerulopathy (LPG) (OMIM: 611771) has been characterized as a rare glomerular disorder leading to nephrotic syndrome and/or kidney failure (2). LPG is characterized clinically by proteinuria and elevated concentrations of triglyceride-rich lipoproteins and their remnants, and histologically characterized by lamellated lipoprotein thrombi in glomerular capillary lumina lacking foam cells. The familial occurrence of LPG has been frequently recognized. LPG is primarily associated with heterozygous *APOE* mutations in the low-density lipoprotein–receptor binding site or around it (3). As a “Mendelian disease” caused by a “single gene” with dominant inherited disease of incomplete penetrance, it also provides a disease model to explore pathogenic roles of *APOE* in some common diseases, such as Alzheimer's disease, type III hyperlipoproteinemia (HLP), and coronary artery disease (2). Trending evidence suggests that *APOE* gene mutations play an important role by potentially increasing the affinity of lipoproteins for the glomerular capillary wall or by enhancing the tendency of mutant apolipoproteins to form aggregates when concentrated. If left untreated, the disease usually progresses to end-stage kidney disease. Lipid-lowering medications, especially fibrates, were found to improve both clinical manifestations and histological alterations. In recent years, there has been a rapid increase in the number of LPG cases reported, and some progress in research into the mechanism and animal models of LPG. We consequently need to update recent epidemiological studies and the molecular mechanisms of LPG. Using “lipoprotein glomerulopathy” or “lipoprotein nephropathy” as key words, we retrieved data from the PubMed, Wanfang (China National Knowledge Infrastructure) and J-STAGE (online platform for Japanese academic journals) databases for literature review (Figure 1). The purpose of this review is to update the epidemiology and clinical features of lipoprotein glomerulopathy, discuss its pathogenesis, summarize current therapeutic options, and present personal perspectives for future research.

## History of LPG

**Figure 2:**
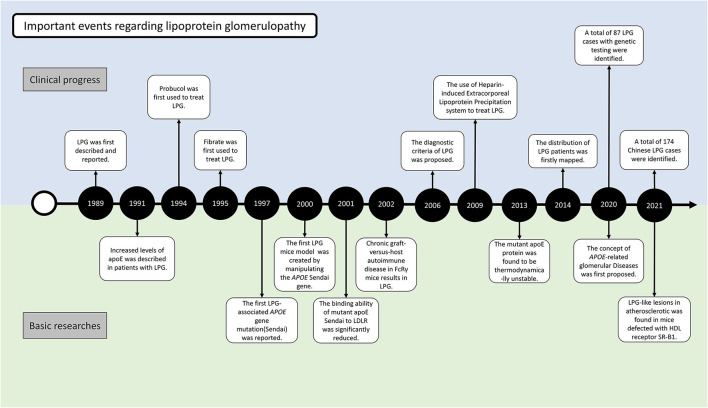
Milestone of LPG research in clinical and basic aspects.

At the 1988 annual meeting of the Japanese Society of Nephrology, Saito et al. first reported the cases of 2 patients who had similar clinical features of renal impairment and glomerular capillary lipoprotein deposition (4) (Figure 2). In 1989, he described the case in the English literature for the first time. The absence of involvement of other organs and the characteristic morphology of the renal lesions could clearly distinguish this disease from other disorders of lipid metabolism. With the presence of lipoproteins in the glomerular deposits and abnormalities in serum lipid levels resembling the pattern observed in type III hyperlipoproteinemia (HLP), this disorder was named “lipoprotein glomerulopathy.” Type III HLP is a condition characterized by the elevation of both cholesterol and triglycerides, accumulation of incompletely catabolized triglyceride-rich lipoproteins, plamar xanthoma, and rapidly progressive atherosclerosis (5). Type III HLP has always been found in individuals who are homozygous for apoE mutations (apoE2/2) and rare in heterozygous state. In 1991, Oikawa et al. reported abnormally elevated levels of apoE in patients with LPG (6). In the report, the levels of lipoprotein components, plasma apolipoprotein profiles, and apoE isoforms were checked in 6 patients. Common features included proteinuria (1.6–10 g/d), normal lecithin-cholesterol acyltransferase (LCAT) activity, type III HLP-like lipoprotein profiles, and significantly higher levels of plasma apoE (>10 mg/dL) compared with the control patients with hyperlipidemic nephrotic syndrome without lipoprotein thrombi, or type IIb hyperlipoproteinemia without renal disease. All the patients had rare apoE isoform patterns (E2/3 in five cases and E4/4 in one case). These findings showed the first evidence that apoE hyperlipoproteinemia was associated with the apoE isoform and lipoprotein metabolic derangement. Familial occurrence of LPG was later recognized, as introduced in the below epidemiology section (7, 8). It was suggested that LPG may be an inherited disease in which abnormal lipoproteins composed of *APOE* mutants accumulate within the glomeruli. This finding was supported by observations of mutations named *APOE* Sendai (Arg145Cys) in 1997 and *APOE* Kyoto (Arg25Cys) in 1998. A total of 17 *APOE* variants associated with LPG have been identified to date, highlighting DNA analysis of the *APOE* gene as one of the most important tools for identifying LPG. These 17 mutations were retrieved from published case reports by manually literature searches, among which 13 have been included in the HGMD database (including *APOE* Kyoto, Tokyo-Maebashi, Sendai, Guangzhou, Okayama, Modena, Las Vegas, Osaka or Kurashiki, Hong Kong, Chicago, Tsukuba, E1, and Kanto, Additional File 1).

Although it was initially assumed that this disease was restricted to East Asia, several cases have been reported in individuals of European ancestry. With the increased awareness of this disease, several patients with nephrotic syndrome of previously “unrecognized” cause have been grouped into LPG in recent years. The total number of reported LPG cases increased to over 200 hundred (details below).

## Epidemiology

The total number of patients was estimated to be <80 in 2011 (9). Due to increased awareness and the advance of genetic testing technology, the number of cases increased to ~150 by June 2019 (10). By an updated literature review, we estimated that the reported number would be at least 274 up to the beginning of the year 2022.

**Figure 3:**
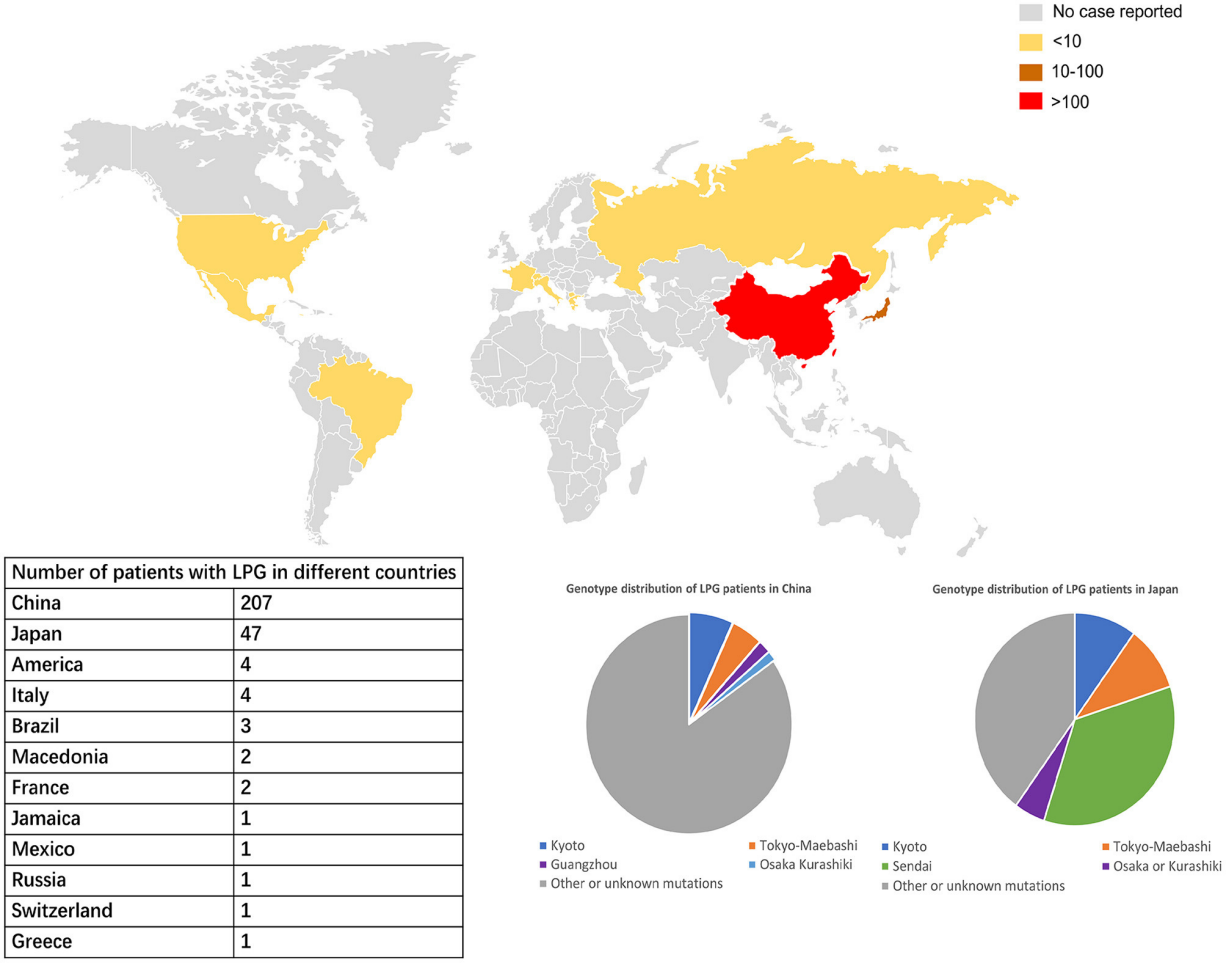
Worldwide distribution of LPG cases. China and Japan are the two countries with the most reported cases of LPG, but the spectra of *APOE* mutation in patients in these two countries are different. In China, *APOE* Kyoto is the major mutant, while in Japan, *APOE* Sendai is the most commonly one. The *APOE* Sendai mutation has not been reported in China thus far.

**Figure 4:**
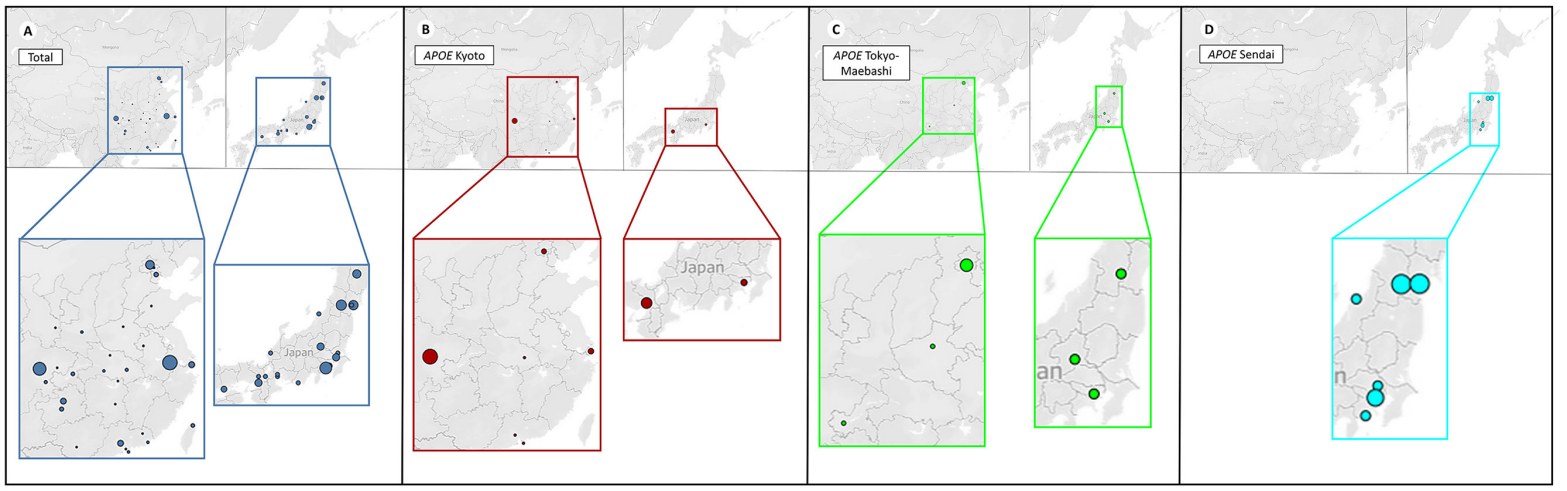
Distribution of patients with different *APOE* mutations. The four plots represent the distribution of patients with different *APOE* mutations in China (left) and Japan (right). For better illustration, each image is shown at the bottom enlarged. The size of each circle represents the number count of people. It can be observed that geographical clustering of LPG cases according to *APOE* mutation types, suggesting a founder effect. **(A)** Patients with LPG were mainly concentrated in the southwestern and southeastern regions in China, and the central and northeastern regions in Japan. **(B)** *APOE* Kyoto was frequently found in southwestern China, and south Japan. **(C)** *APOE* Tokyo-Maebashi was dominant in cases from Beijing in China, and the central Japan. **(D)** *APOE* Sendai was not reported in China, and distributed most in central Japan.

LPG showed significant regional and familial aggregation. The majority of cases were reported in Japan and China (Figures 3, 4). It is mainly distributed in southwestern and southeastern China and in central and north-central Japan (11). Some hot spots of gene mutations have been described, suggesting a founder effect of the gene mutation. *APOE* Sendai (Arg145Pro) was mainly observed in north-central Japan, particularly in Yamagata and Miyagi (12), whereas it has not been reported in China. *APOE* Kyoto (Arg25Cys) was reported as the most frequent mutation in LPG throughout the world, including in southwestern China, Japan, France and the USA. However, a report in 2014 showed that 35 patients within 31 unrelated Han families with biopsy-proven LPG resided in a small county of Sichuan Basin in southwest China, indicating regional clustering with the same genetic background. It was thus suggested that the descendants of *APOE* Kyoto in this area were derived from a single founder. In contrast, *APOE* Guangzhou and Tokyo-Maebashi were reported to be dominant in cases from the southeast area of China (7, 8, 11, 13). The association of *APOE* Kyoto between the Sichuan Basin and other areas is still difficult to explain. It may be possible that *APOE* Kyoto spread from China across multiple countries worldwide because it is historically known that the international population mobility of Chinese was more frequent than that of Japanese in ancient times (11). This theory may be similar to the idea that LPG cases may have emigrated along with tea culture to expand from China (11). Several other *APOE* variants have recently been reported in Western countries, including the United States, Russia, Italy, Brazil, and France. Other *APOE* variants associated with LPG have been detected across the world (14–20). Although many of the reported cases were reported to be sporadic, some studies reported familial clustering, supporting its genetic component with a single gene inheritance mode (21–23). But perhaps most of the family members lack of symptomatic signs or genetic testing, making them undiagnosed. No heritability studies have been documented to date. One of the largest pedigree was reported in 2008, in which 5 cases in 3 generations were observed (8). Another large pedigree with 3 cases in 2 generations was reported in 2014, together with additional 17 pedigrees with LPG (7).

Apart from humans, interestingly, an observational study in 2016 reported that a captive squirrel (Sciurus vulgaris) spontaneously developed LPG-like disease. This was the first time that LPG-like disease was observed in an organism other than humans. The kidney pathology of squirrels is similar to that of human LPG, but genetic mutations have not been determined (24).

As the majority of the reports on the epidemiology of LPG were based on case reports, to systemically update this information, we conducted a literature review using the search item “lipoprotein glomerulopathy” from various databases (PubMed, China National Knowledge Infrastructure, and J-STAGE) from 1988 to January 2022. A total of 274 LPG cases were identified, and the epidemiological details can be found in Additional File 1.

According to updated statistics, China and Japan have a comparatively higher prevalence of LPG, with 207 and 47 patients, respectively. There is still lack of precise epidemiological studies in LPG. Based on the current meta data from literature review, it was estimated that the prevalence of LPG is about 3.74 per 10 million in Japan and 1.43 per 10 million in China. Surely, it may be underestimated. As mentioned above, the areas most affected by LPG were located in southwestern and southeastern China and central and northeastern Japan. A total of 20 cases have been reported in other countries and territories. Among all the cases, there were 137 males and 137 females, with an average age of diagnosis of 35 years old (from 7 to 72 years). In terms of age of onset, a total of 188 patients had documented information. There were 8, 23, 112, 42 and 3 cases with age of onset ≤ 10, 11–17, 18–45, 46–65, and >65. It suggested that about 60% of the cases were diagnosed in young adults (18–45 years old) (13).

With regard to specific mutation distribution, *APOE* Kyoto, *APOE* Tokyo-Maebashi and *APOE* Sendai are the three major observed forms, with 53, 13, and 14 reported patients, respectively. In China, the Sendai mutant has not yet been reported. The Kyoto mutant is mainly diagnosed in Sichuan Province in southwest China, while the Tokyo-Maebashi variant is mainly diagnosed in Beijing, suggesting that LPG patients in the same region may share a common genetic ancestor; demonstrating the “founder effect.”

## Pathogenesis

The precise pathogenesis of LPG is still not well-understood. Several lines of evidence suggest that alterations in apoE structure and function play a fundamental role in the pathogenesis of LPG. It has long been hypothesized that defective lipoproteins are prone to deposit in the kidney. Indeed, all the patients reported to date were found to have heterozygous mutations in the *APOE* gene, along with elevated serum concentrations of apoE lipoproteins and the presence of apoE in the glomerular deposits. Disturbances in kidney structure or function may also be pivotal in the formation of lipoprotein aggregates, as the disease is kidney specific without other organs obviously affected. However, the recurrence of LPG after renal transplantation suggests that renal abnormalities may not be necessary for the development of the disease.

### *APOE* Gene and apoE Function

**Figure 5:**
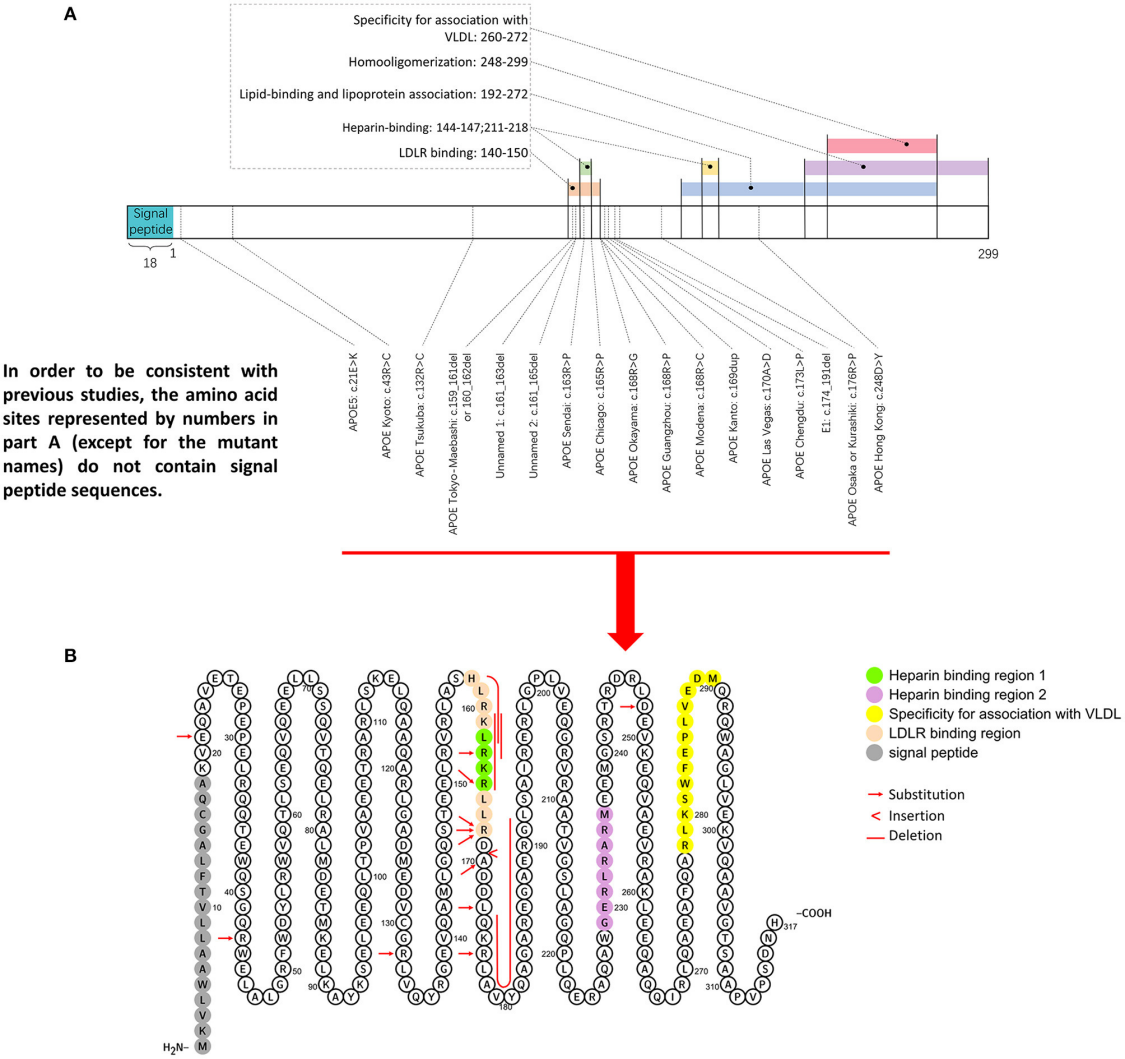
Representative mutation distributions of the apoE protein. **(A)** Seventeen mutations of the *APOE* gene leading to LPG have been located, most of which are concentrated at amino acid sites 140–180. This region contains various important apoE functional domains, including the LDLR binding domain and HSPG binding domain. “Hot spot” mutations suggested that the change in the binding ability of the apoE mutant to LDLR and HSPG is an important factor in LPG pathogenesis. **(B)** *ApoE* mutation sites were labeled in the amino acid sequence diagram of apoE protein. It showed that hot spot of apoE mutation is among AA 140–160.

The *APOE* gene is located on chromosome 19q13.2 and comprises 4 exons with 3,603 base pairs, which are evolutionarily conserved in a variety of terrestrial and marine vertebrates (25) (Figure 5). The apoE protein is a 34 kDa circulating glycoprotein of 299 amino acids, with an additional 18 amino acids as a signal peptide. This protein can be synthesized by several cell types, in which hepatocytes account for the majority. High quantities can also be observed in brain, i.e., by astrocytes and glial cells in the cerebral cortex and by neurons in the frontal cortex and hippocampus (26).

The amino acid sequence of apoE can be simply divided into three parts: the N-terminal (AA 1–199), the hinge region (AA 200–215), and the C-terminal (AA 216–299). There are two important binding regions in the N-terminal sequence that have overlapping gene sequences, the low density lipoprotein receptor (LDLR) binding region (27, 28) (142–150) (29) and the heparin sulfate glycoprotein (144–147) binding region (30). Most *APOE* mutations associated with LPG are observed in these two regions. Other regions with functional significance included four helix regions (AA 20–160), a lipid insertion sequence (244–272) and a homo-oligomerization region (248–299) (31–34), which have been investigated less.

**Figure 6:**
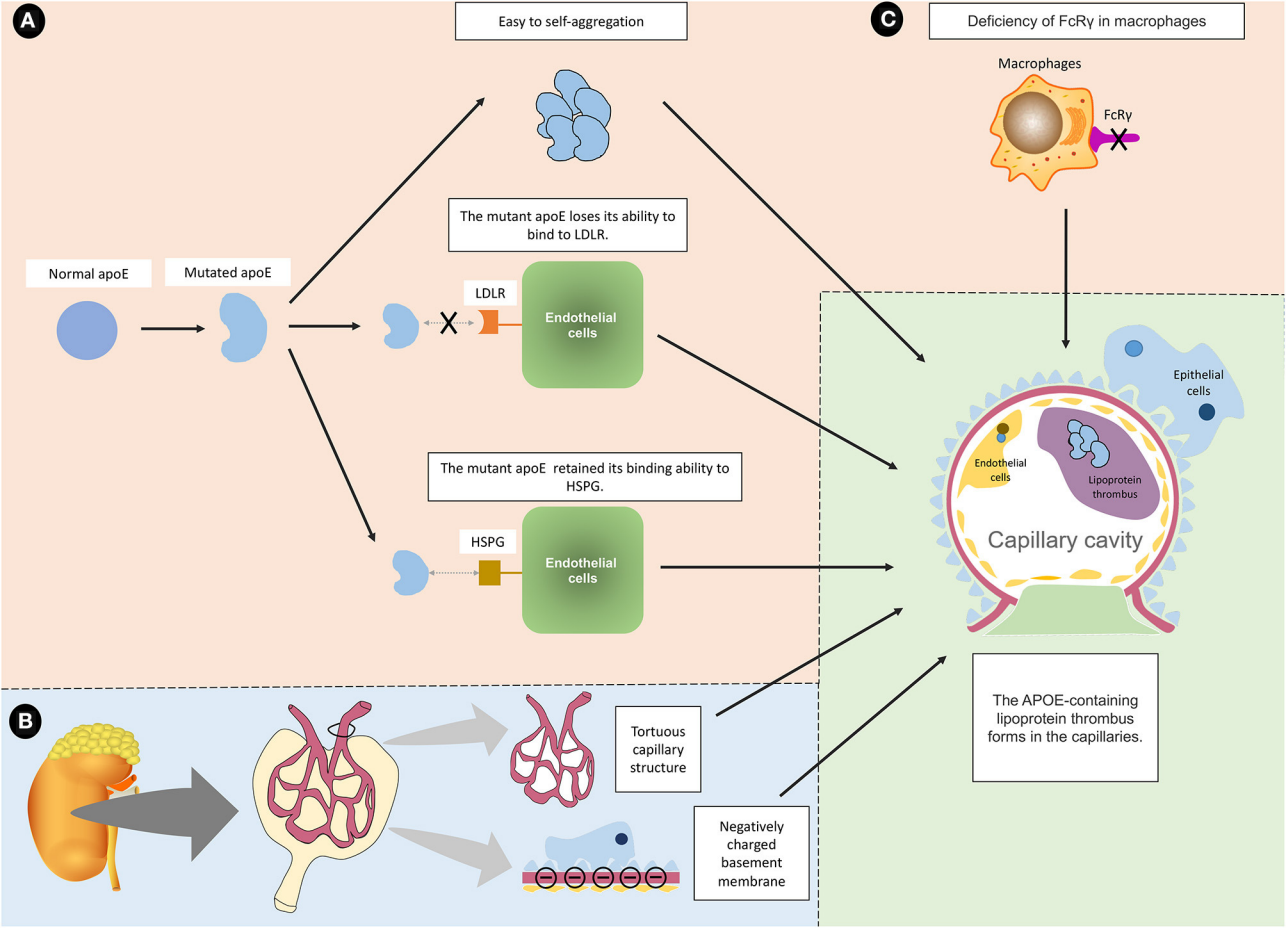
Summary and proposed mechanisms of LPG. **(A)** The mutated apoE protein is known to cause LPG through three main mechanisms. (a) The mutant protein tends to aggregate, and the aggregated macromolecules are more likely to drive the formation of lipoprotein thrombi. (b) The mutant protein loses its ability to bind to LDLR, making it difficult to eliminate. (c) The mutated protein retains the ability to bind to HSPG, which allows it to bind to endothelial cells. For the above reasons, the mutated apoE protein aggregates into macromolecules on the surface of endothelial cells without being cleared. **(B)** In addition to APOE mutation, it is speculated that intrinsic glomerular features may interact with apoE variants and lipoprotein abnormalities, which exacerbate the induction of LPG. For example, tortuous glomerular capillaries are conducive to the formation of thrombi. The negatively charged base membrane and the positively charged mutant protein may attract each other. **(C)** FcRγ deficiency may also lead to LPG because it affects the phagocytic function of macrophages.

As the ligand for the LDL receptor family and heparan sulfate proteoglycans (HSPG), apoE associates with triglyceride-rich lipoproteins in mediating clearance of their remnants (Figure 6). When exogenous lipids enter the bloodstream from intestinal villi, new chylomicrons are formed to obtain apoC and apoE from high-density lipoprotein (HDL). Some chylomicrons are utilized by body tissues, while the remaining chylomicrons enter the liver through interactions with apoE and LDL receptor-associated proteins (LRP).

ApoE is also important in endogenous lipid metabolism. Hepatocytes secrete VLDL containing apoE, and these VLDL are converted into IDL. The subsequent metabolism of IDL can be divided into two pathways, through interactions between apoE and LRP or metabolism into LDL (35–37). The degradation of both IDL and VLDL is partially dependent on apoE. This may explain why their concentrations are elevated in the blood of patients with LPG.

### *APOE* Mutations in LPG

Two coding variants in the human *APOE* gene, rs429358 (Cys130Arg) and rs7412 (Arg176Cys), define the apoE protein as three isoforms (E2, E3, and E4) (38), among which E3 is the most common isoform, and they are different in the amino acid residues at residues 112 and 158: both cysteine—apoE2, both arginine—apoE4, and one cysteine—apoE3 (the wild type). It has been confirmed that different subtypes are associated with different predispositions to human diseases (39). The affinity between the E2 subtype and the low-density lipoprotein receptor (LDLR) is only 1% of that of the E3 subtype, resulting in lipid clearance disturbances and type III HLP. The E4 subtype is mainly associated with diseases of the central nervous system, such as Alzheimer's disease (34, 40–42). However, in LPG, the most common subtype was reported to be E3/E3 or E3/E4 (3, 9).

In 1991, abnormally elevated levels of apoE were observed in patients with LPG for the first time (6), followed by the discovery of the mutation *APOE* Sendai in 1997. Further *APOE* mutation has been suggested to be the most important etiologic factor in the pathogenesis of LPG (43, 44). Supporting evidence was obtained from animal models. In 2000, by introducing the *APOE* Sendai mutation into *APOE*-deficient mice, it was observed that both increased lipid levels and LPG-like renal pathology in the mice (45). Several other variants of *APOE* associated with LPG have been identified since then. A similar cause-effect of the *APOE* Kyoto mutation in LPG can also be observed in *APOE*-deficient mice, further supporting that a single gene mutation can cause LPG (46).

To date, a total of 17 *APOE* gene variants associated with LPG have been reported. Eight of these mutations reside in the LDLR binding region (140–150 sites), among which 5 reside in the HSPG binding region (144–147 sites). Another hot spot is located in the region spanning AA 150–180, where 5 mutations have been reported (25, 33), but few functional studies have been conducted. More recently, a case report suggesting a 28-year-old man presenting with severe proteinuria and hyperlipidemia had compound heterogeneous mutations of the *APOE* gene inherited from his mother (p.Arg50His) and father (*APOE* Kyoto). Each his parents with a heterogeneous mutation had normal kidney function without proteinuria (47). This is the first time that the combination of the 2 mutations was identified in the same case as an autosomal recessive genetic disorder. It seemed that the case showed much severe phenotype. But further precise investigation on both genetic and disease mechanisms will be needed. Since the patient carried *APOE* Kyoto mutation, and the new p.Arg50His variant has not been verified functionally pathogenic, therefore, this mutation is not listed in our pathogenic mutations of LPG. We assume that a dominant effect of *APOE* Kyoto cannot be totally rule out. Apart from this, a case was reported to have a combination of *APOE* Kyoto and *APOE* Hong Kong (Asp230Tyr) mutations (48). But as co-segregation analysis was not taken, it was difficult to confirm the significance of the respective mutation. There was also a case report of a 51-year-old Japanese woman who had 2 mutations within the same allele (a combination of *APOE* Chicago (Arg147Pro) and *APOE*5 (Glu3Lys) inherited from her mother. But her mother did not have any phenotypes (49). These data suggested LPG may be far complicated than previously speculated. Additional genetic studies may be needed, i.e., from a hypothesis-free whole genome sequencing in large cohorts.

### ApoE Mutation-Related Factors Leading to LPG

#### Reduced Structural Stability of apoE Protein

Normal apoE protein is highly helical and has the capability to be transformed between different tertiary structures to bind lipids or proteins. In 2013, it was found that different mutated proteins [*APOE* Chicago (Arg147Pro), *APOE* Sendai (Arg145Pro), *APOE* Osaka or Kurashiki (Arg158Pro)] showed different protein structural stabilities (50). Of note, the common resultant characteristic of these three mutations was that arginine was replaced by a proline residue. It is universally suggested that proline residues may breakdown a transmembrane helix. Insertion of a proline residue in the middle of an α-helix is known to destabilize it by over 3 kcal/mol, effectively disrupting the helix (51, 52). These mutated apoE proteins all exhibited reduced helicity, leading to decreased structural stability evidenced as protein denaturation even at the physiological temperature of 37°C. When the hydrophobic surface is exposed, apoE may be more prone to aggregate and form large lipoprotein granules (50). Along with this idea, it was also confirmed that similar structural and functional changes can be observed in three other non-proline-substituted mutants [*APOE* Kyoto (Arg25Cys), *APOE* Tsukuba (Arg114Cys), and *APOE* Las Vegas (Ala152Asp)] (53). *In vitro*, all three mutated proteins showed decreased stability and an increased tendency to aggregate. Likewise, a study in Alzheimer's disease showed that the decreased structural stability of apoE may contribute to the formation of neurotoxic fibrils (54).

#### Reduced apoE Binding Capacity to Different Receptors

ApoE mutations may also contribute to reduced binding capacities to receptors (55, 56). It was observed that the binding ability of apoE Kyoto and apoE Sendai to LDLR was significantly reduced to just 10 and <5%, respectively, compared to the wild type (57, 58). The decrease in the apoE binding ability contributes to impaired degradation of lipoprotein and accumulation. This is consistent with the observation that hyperlipidemia is common in LPG, especially with increased VLDL and IDL. However, the binding capacity of apoE2 to LDLR was <1% of the normal value (33, 59), while apoE2-induced type III HLP only had dyslipidemia without pathological kidney changes, indicating that impaired LDLR binding abilities may not be the determining factor in kidney damage. Although the *APOE* Sendai mutation decreased the binding ability of apoE with LDLR to <5%, it decreased its binding ability with HSPG to 66% (57). It was suggested that the retained HSPG binding activity could enable apoE-rich lipoproteins to enter and attach to the Disse space (60, 61), which is pivotal in the initial rapid clearance step for lipoproteins. Heparan sulfate proteoglycans (HSPGs), which are also abundant in the space of Disse, may play an important role in mediating this enhanced binding. When apoE arrived and aggregated in the Disse space, they would normally enter the liver through interaction with LDLR, but the mutated apoE lost the LDLR binding ability, so they could not enter the liver to be metabolized (62, 63). Since HSPG is highly expressed in the glomerular basement membrane (64, 65), it may play a role in the renal deposition of lipoprotein. Concordant with this hypothesis, evidence has shown that the binding ability of products is enhanced in apoE Chicago to glomerular capillaries and in apoE Kyoto to human umbilical vein endothelial cells (16, 66).

### Other Predisposing or Synergic Factors Leading to LPG

#### Functional Deficiency of the Fc Gamma Receptor (FcγR) and Macrophages

As some individuals carrying *APOE* mutations are asymptomatic, it is speculated that some other factors may further contribute to LPG (21). Two clinical reports noted the recurrence of LPG in the transplanted kidney. Sustained inflammation induced by chronic graft-vs. host disease (GVHD) might be a predisposing factor (19, 67, 68). By injecting donor spleen cells into recipient mice for 2–4 months, it was observed that GVHD could induce LPG-like changes in Fc receptor gamma chain (FcRγ)-deficient mice, with hematuria, proteinuria, renal capillary lumen thrombosis and interstitial mononuclear cell infiltration. It was shown that macrophages from FcRγ-deficient mice had a decreased ability to clear LDL (69). Macrophages possess several different pathways in recognition and clearance of modified (oxidized) LDL, including scavenger receptors and FcRs (70, 71). Supporting this, a drastic decline of scavenger receptor CD36 was observed in LPG. It was speculated that partial reduction of modified (oxidized) LDL uptake by macrophages could result in the lipoprotein deposition in the kidney during the long course of chronic GVHD.

More direct evidence was obtained from humanized mice. When the *APOE*3 genotype or *APOE* Sendai mutation was introduced into FcRγ and *APOE* double-knockout mice, both strains of mice developed lipoprotein thrombosis, and the mice with *APOE*3 showed more lipoprotein thrombus in the kidney than those with the *APOE* Sendai genotype. This observation was absent in mice with wild-type *APOE*. Introduction of *APOE*3 in both FcRγ-deficient mice and FcRγ wild-type mice showed lipoprotein thrombosis, whereas the phenotype was much more severe in FcRγ-deficient mice (72). These results indicated macrophage impairments derived from FcRγ deficiency (73–75) were insufficient for the development of LPG, since the FcRγ deficient mice with normal murine apoE showed no lipoprotein thrombosis. However, under conditions with xenogeneic apoE, especially human apoE3, the FcRγ deficient mice may develop severe LPG.

It was also observed that a small amount of apoE can be produced by macrophages, which was considered to play a role in suppressing hyperlipidemia and arteriosclerosis. Because it was showed that expression of macrophages producing apoE Sendai in mice that received a bone marrow transplant protected against atherosclerosis while induced LPG (76–78). It was thus suggested that macrophages may play various roles in apoE related lipoprotein metabolism. Both hyperactivity or suppression can be an important factor in different types of renal lipidosis. LPG depend upon suppression of macrophages. ApoE derived from macrophages is affected by its mutation and may regulate disease activity. Functional studies are still needed in the future.

#### Renal Intrinsic Factors May Promote Glomerular Lipoprotein Deposition

The above data is insufficient to explain why lipoprotein is specifically deposited in glomerular capillaries in LPG, which is different from other diseases such as atherosclerosis. In particular, because lesions are localized to glomeruli, intrinsic glomerular factors may interact with apoE variants and lipoprotein abnormalities to induce LPG. There might be some special factors in the glomeruli. Mutated apoE protein may present different electric charges compared to normal proteins, and therefore, they exhibit a higher affinity for negatively charged glomerular basement membranes (9). It is suggested that the presence of highly conserved acidic residues within the lipoprotein receptor (LR) modules and the positively charged region of apoE (residues 136–150) may support the hypothesis that ligand–receptor recognition is due to electrostatic interactions, so a change in electron charge might enhance the bonding (79). The tortuous structure of glomerular capillaries might also contribute to lipoprotein deposition.

#### Oxidative Stress May Dampen Kidney Damage

It has long been believed that hyperlipidemia may play a detrimental role in kidney pathology directly or indirectly through inflammation, ROS production, endogenous electrical stress and other pathways (80, 81). Among these pathways, oxidative stress may be of special importance, as carboxymethyllysine (CML), hydroxynonenal (HNE)-protein, and malondialdehyde (MDA)-lysine were reported to be found in the kidney of a patient with LPG (68). These substances are the products of lipid peroxidation. These aldehydes may cross-link covalently with matrix tissue proteins and further alter structure and function. They may also have direct impact on parenchymal cells by cross-linking cell surface proteins to reduce intracellular responses (82–84). As the antioxidant domain of *APOE* overlaps with the LDLR binding region (85), mutations in the LDLR binding domain may shed unfavorable effects on its antioxidant capacity. Moreover, the roles of oxidative stress and hyperlipidemia in LPG are indirectly supported by clinical evidence that some patients experience remission after treatment with the antioxidant probucol and the lipid-lowering drug fenofibrate (10, 86).

#### Other Mechanisms

There have been reports that no *APOE* mutations were found in a number of LPG patients (87). Possible explanations include changes in other unknown genes, mutations in introns or regulatory sequences, epigenetics, and environmental influences.

For example, a recent study showed that a defect in HDL receptor named scavenger receptor class B type 1 (SR-B1) was associated with LPG-like lesions in atherosclerotic mice, the severity of which can be alleviated using probucol (88).

Factors affecting CKD progression, such as fibrosis, apoptosis, local tissue injury, and infiltration of immune cells, may also be involved in LPG.

These data support a complex etiology of LPG, and additional pathogenic factors warrant further elucidation.

## Clinical and Pathological Manifestations

### Diagnostic Criteria Proposed by Japanese Nephrologists

In 2006, a single set of diagnostic criteria for LPG was proposed by Japanese nephrologists (2). The criteria were mainly based on four items.

- different levels of proteinuria;
- dilatation of glomerular capillary lumina with pale-stained substances on light microscopy;
- fingerprint-like concentric lamellar structure in electron microscopy;
- Type III HLPs with high apoE concentrations are usually associated with a heterozygous apoE phenotype, E2/3 or E2/4, by means of isoelectric focusing electrophoresis (IEF), but sometimes with an uncommon type, e.g., E1/3 or others.

It was suggested that although genetic testing was not necessary for definitive diagnosis, it should be performed in patients with suspected LPG whenever possible to confirm the diagnosis.

### Clinical Manifestation of Glomerular Disease Is Always Non-specific

Initially, LPG may cause no symptoms. Symptoms and signs are due to the buildup of waste products and fluid retention in the body (49, 89, 90). In general, nephrotic syndrome may be present in ~70% of patients (91). However, the levels of proteinuria may vary significantly among different patients. The 24 h urinary protein levels reported ranged from 0.5 to 24 g/24 h (92, 93). Without proper treatment, CKD will progress (2, 14, 94–96), and approximately half of patients will develop end-stage kidney disease over 1–27 years.

### Elevated Serum ApoE and Hyperlipidemia

One of the most characteristic laboratory indicators of LPG is elevated serum apoE levels. It was reported that patients with LPG had a mean serum apoE concentration of 11.14–17.1 mg/dL (3.9–71.0 mg/dL), approximately twice the upper level of the normal population (2, 7, 23, 97, 98).

Another characteristic is overt dyslipidemia, with a predominance of triglycerides, mostly >6 mmol/L (15, 16, 91). In a few cases, VLDL levels were reported to be elevated (~4–6 mmol/L) (99, 100). Hyperlipidemia in LPG is similar to that in familial type III HLP. Type III HLP, first recognized in 1967, is caused by homozygous apoE2 mutations. The mutation was reported to weaken the binding force between apoE2 and LDL receptors (101, 102). Although both diseases are associated with the *APOE* gene, LPG and type III HLP show obvious differences. In type III HLP, atherosclerotic cardiovascular disease and xanthomatosis are common (103, 104), but they are rare in LPG.

### Great Phenotypic Heterogeneity and Genotype-Phenotype Correlations

We further checked the clinical manifestations of LPG by systematic literature review. An additional file shows this in more detail (see Additional File 2). The mean value of plasma albumin for the reported cases was 29.3 g/L (SD = 6.2 g/L, ranging from 12 to 47 g/L), and the mean 24-h urinary protein level was 4.5 g/d (SD = 3.4 g/d, ranging from 0.8 to 24 g/d). Nephrotic range proteinuria and nephrotic syndrome were key features. The mean creatinine was 129.2 μmol/L (SD = 177.5 μmol/L, and ranging from 21.2 to 1859 μmol/L), the mean urea nitrogen was 8.1 mmol/L (SD = 5.52 mmol/L, and ranging from 2.7 to 39.9 mmol/L), and the mean eGFR was 81.6 ml/min/1.73 m^2^ (SD = 32.5 ml/min/1.73 m^2^). Of these patients, 38.7% had CKD1, 35.5% had CKD2, 18.7% had CKD3, 3.2% had CKD4, and 3.9% had CKD5. In terms of blood lipids, the average triglyceride level of these patients was 3.4 mmol/L (SD = 2.0 mmol/L, ranging from 0.7 to 20.6 mmol/L) (reference range 0.6–1.7 mmol/L), which is approximately twice the normal upper limit. Total cholesterol was 6.8 mmol/L (SD = 2.4 mmol/L, and ranging from 2.8 to 22.9 mmol/L) (reference range 2.8–5.2 mmol/L), LDL was 3.6 mmol/L (SD = 1.4 mmol/L, and ranging from 1.2 to 10.6 mmol/L) (reference range 2.1–3.1 mmol/L), and HDL was 1.4 mmol/L (SD = 0.7 mmol/L, ranging from 0.5 to 8.8 mmol/L) (reference range 0.9–1.6 mmol/L). Most of the patients had significantly elevated serum apoE, with a mean of 12.1 mg/dL (SD = 6.7 mg/dL, and ranging from 3.1 to 42.3 mg/dL). Great fluctuations of standard deviations suggested great clinical heterogeneity of LPG.

We collated the clinical information of the cases with the top four *APOE* genotypes (Kyoto, Tokyo-Maebashi, Sendai and Osaka or Kurashiki) (Table 1). The age of onset for patients with *APOE* Tokyo-Maebashi seemed to be younger than that of other mutants. Patients with *APOE* Sendai appeared to have the lowest urinary protein and blood lipid levels. Patients with *APOE* Kyoto had the most severe renal manifestations. There was few clinical prognostic information available for LPG. However, according to literature review and case reports, some of the cases with LPG will progress to ESKD. These reported ESKD caused by LPG can be observed in several *APOE* genotypes including Kyoto, Tokyo-Maebashi, Osaka or Kurashiki, Guangzhou, and Sendai. And the reported *APOE* phenotypes were E2/3 and E3/3 (7, 8, 105–107). Thus, it still difficult to predict prognosis based on mutation types.

**Table 1:** Genotype and phenotype information based on meta data.

	***APOE*** ** Kyoto**	***APOE*** ** Tokyo-Maebashi**	***APOE*** ** Sendai**	***APOE*** ** Osaka or Kurashiki**	***P*** **-value**
Total number of cases	53	15	13	6	
Age	38.1 (34.5–38.1) (*n* = 53)	20.0 (10–41) (*n* = 15)	31.7 ± 18.9 (*n* = 13)	32.0 ± 8.7 (*n* = 6)	0.142
Albumin (g/L)	26.4 (26.4–26.4) (*n* = 48)	31.5 ± 9.1 (*n* = 16)	NA	27.1 ± 9.4 (*n* = 5)	0.024
Serum creatinine (μmol/L)	98.1 (98.1–98.1) (*n* = 46)	72.0 (43.5–90.5) (*n* = 13)	79.6 (37.1–256.4) (*n* = 5)	NA	0.000
eGFR	79.0 (79.0–79.0) (*n* = 46)	82.0 (59.7–156.5) (*n* = 15)	91.9 ± 27.0 (*n* = 12)	105.7 ± 27.1 (*n* = 5)	0.303
TC (mmol/L)	7.0 (7.0–7.0) (*n* = 53)	6.0 (4.9–7.1) (*n* = 14)	5.8 ± 1.6 (*n* = 13)	5.3 ± 2.8 (*n* = 6)	0.012
TG (mmol/L)	3.5 (3.5–3.5) (*n* = 53)	2.8 ± 1.7 (*n* = 14)	1.7 (1.5–3.8) (*n* = 13)	2.6 ± 1.0 (*n* = 6)	0.031
24 h UPRO (g)	5.1 (3.8–8.8) (*n* = 16)	2.8 ± 1.6 (*n* = 10)	1.6 (1.0–2.2) (*n* = 11)	7.4 ± 4.7 (*n* = 5)	0.006

*Data are presented as mean ± SD for normalized data or median (25^th^-75^th^ percentile) for non-normally distributed data*.

*Due to missing information for some case reports, n in the bracket represents the amount of data*.

*NA means data unavailable*.

### Extrarenal Manifestations and Other Complications

Other complications rarely have been reported. Known comorbidities included splenomegaly, thalassemia, psoriasis, abdominal aortic aneurysm, pleural effusion, and neurofibromatosis type I (14, 67, 99, 108, 109). However, there is no evidence of a direct link between LPG and these diseases. The only related extrarenal manifestation of LPG that has been confirmed is intravascular coagulation (110). It was reported that a 50-year-old white man had severe proteinuria with high lipid levels and a kidney pathology of LPG. He also had hypertension and coronary heart disease. The patient's heart failure was speculated to be due to cardiac amyloidosis secondary to multiple myeloma. For this reason, cardiac biopsies were requested and showed that small blood vessels in the endocardia were filled with eosinophilic substances, which were similar to those in the kidney. A more recent case reported that a 21-year-old Malaysian-Swiss male with LPG developed atypical hemolytic uremic syndrome (aHUS). Aggregation of apoE was suggested by the authors as a risk factor in initiating the occurrence of aHUS in his case (107). Regardless, the etiology was unclear and may be difficult to determine.

### Pathological Manifestations in the Kidney

**Figure 7:**
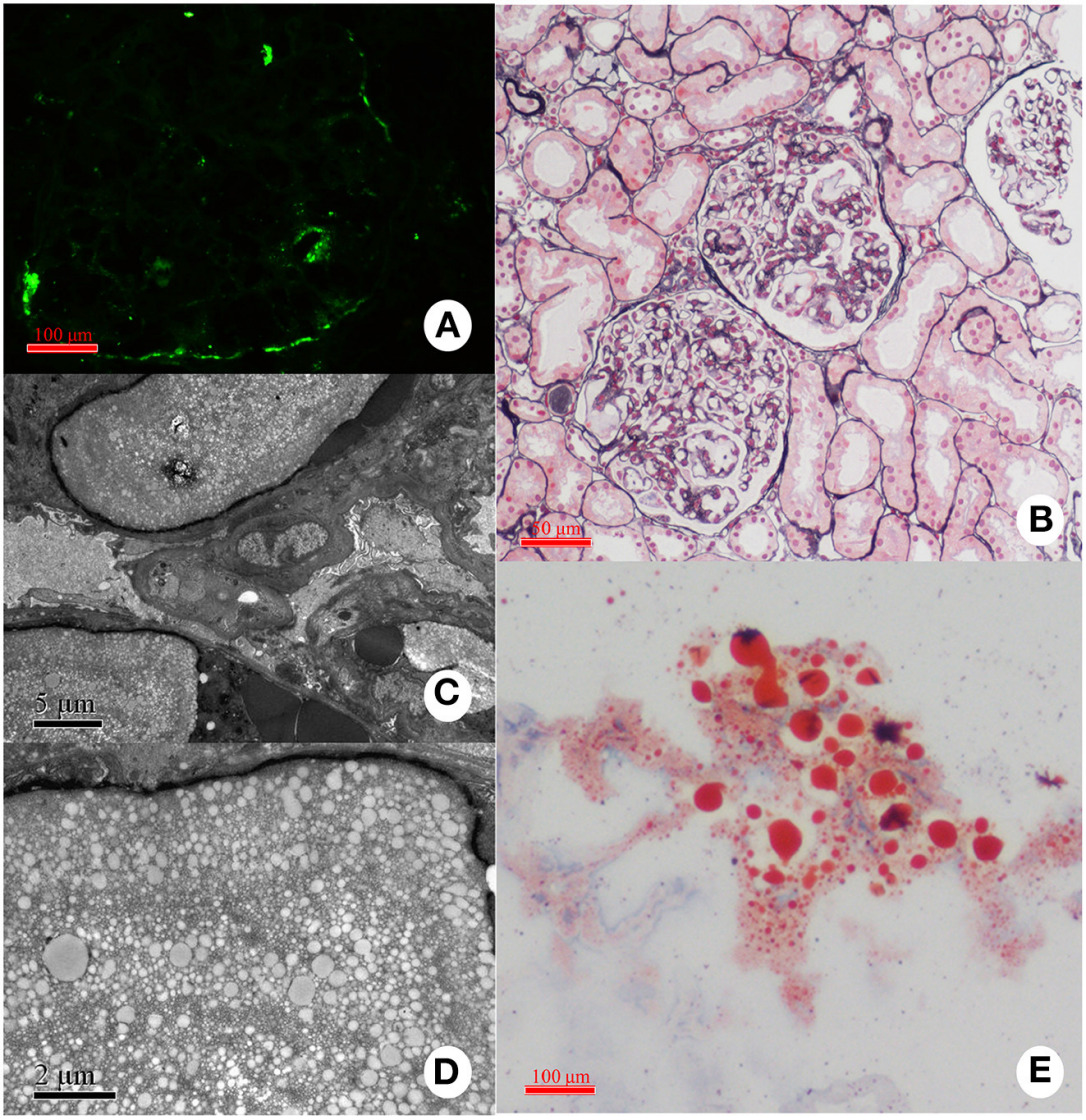
Representative pathological findings in a case with LPG. **(A)** Immunofluorescence study: the deposition of apoE is present mainly in the capillary lumina. **(B)** Light microscopy: Dilated capillary loops exhibiting an eosinophilic lipoprotein thrombus in the capillary lumens. **(C,D)** Electron microscopy: Diffuse foot-process effacement and lamellated fingerprint-like thrombi in capillary lumens, which are composed of granules and vacuoles of various sizes. **(E)** Oil red O staining: Numerous red droplets are seen in the thrombus-like substances in the glomerular capillaries.

The most typical pathological appearance of LPG under light microscopy is the dilatation of the glomerular capillary lumen filled with eosinophilic granular and vacuolar thrombosis (Figure 7). The thrombus is positive for oil red O or Sudan red and negative for silver or PAS staining, indicating the presence of a lipid component in the thrombus (14, 22, 110–112). Using immune-electron microscopy, it has been found that lipids are surrounded by apoE and that the thromboid material is composed of lipoproteins (113). Other manifestations under light microscopy include swelling of endothelial cells and vacuolar degeneration with a small number of lipid droplets in podocytes. In the advanced stages of the disease, the mesangial cells and stroma are thickened, and there may be uneven insertion, leading to thickening of the basement membrane and the formation of the dual-track sign and eventually glomerular sclerosis. Epithelial vacuoles and granular degeneration may be observed in renal tubules in the early stages. Similar to other glomerulopathies, as the disease progresses, renal tubule atrophy, interstitial edema, monocyte infiltration and fibrosis, and thickening of the arteriole wall will be common (21, 114). A study also reported that CD68+ foam cells were present in patients' kidneys, further suggesting that macrophages may be involved in the pathogenesis of LPG.

The most common deposits are apoE and apoB in immunofluorescence, which might be taken as supporting evidence for the diagnosis of LPG. In the previous literature, however, immunoglobulin and complement, such as IgM, IgA, and C3 depositions, may be observed in mesangial and capillary walls in many cases, and scattered C1q deposits were also reported (16, 23, 48, 114, 115). However, most of these depositions were suggested to be non-specific, and no specific pathology pattern has been described until now.

Under electron microscopy, lipoprotein thrombosis is characterized by a fingerprint-like concentric lamellar structure, sometimes referred to as a sand stone structure (2). Some other pathological descriptions include glomerular telangiectasis, filled with protein material covered with lipid vacuoles, and lamellar vacuoles of varying sizes, with a network of vacuoles separated by high electron density bands. Lysosomes of endothelial cells and podocytes were increased, and lipid vacuoles were also found in the cytoplasm and lysosomes. Podocytes showed diffuse and complete foot process effacement with the accumulation of electron-dense material in the mesangium and glomerular basement membrane. The glomerular basement membrane demonstrated focal subcutaneous electron dense deposition (100, 110–112, 114, 116).

Because the main pathological feature of LPG is lipoprotein thrombosis, it should be differentiated from other diseases with thrombotic substances, such as macroglobulinemia nephropathy and cryoglobulinemia nephropathy.

## Treatment

There is still a lack of specific or targeted therapies for LPG. Most of the regimens available were based on lowering proteinuria and hyperlipidemia. In the absence of clinical trials, current data are mostly from case reports or observational studies.

### No Beneficial Effects From Immunosuppressants or Transplantation

Early in the history of LPG, the patient was often treated with glucocorticoids with or without immunosuppressants, similar to other nephrotic syndromes. However, it was soon proven ineffective (43, 95, 108, 117, 118). Additionally, in the twentieth century, it was reported that LPG relapsed in nearly all ESKD patients caused by LPG even with intensive immunosuppressive therapy. Therefore, kidney transplantation and immunosuppressants were not recommended because the culprit was abnormal lipoprotein components in the blood instead of the kidney. However, in some case reports, leflunomide was reported to ameliorate proteinuria in some patients. It was initially used to replace prednisone, which was found to have a complete disappearance of kidney symptoms after leflunomide was added (119). The exact mechanism of leflunomide may not be related to its immunosuppressive effect.

### Regimens Targeting Lipoprotein and Hyperlipidemia

Regimens have achieved some success in supporting the pathologically causal role of lipoprotein and hyperlipidemia in LPG. The first drug was probucol, which reduces total TC and LDL levels and is widely used in the treatment of hypercholesterolemia (120). Probucol is also an antioxidant, and it may mitigate glomerular damage caused by oxidative stress of mutated proteins. As early as 1994, it was used in an LPG female case of 54 years old, effectively reducing hyperlipidemia, proteinuria and the complete elimination of her glomerular lipoprotein thrombosis (86). Later, different case reports confirmed the efficacy of probucol therapy (116, 121).

Fenofibrate and benzafibrate can significantly reduce blood VLDL levels by acting on peroxisomal proliferator-activated receptor (PPAR) and activating lipoprotein lipase (LPL) and thus reducing TG and LDL. A small clinical contrast study based on 35 patients supported the efficacy of fibrates in LPG. After 12 months of treatment, their lipid profiles, proteinuria, and serum albumin improved, and their serum apoE decreased significantly with stable renal function (7, 14, 93, 121, 122).

Niceritrol is a nicotinic acid derivative that has been used to lower lipoprotein levels and reduce proteinuria in patients with chronic kidney disease associated with hyperlipidemia (123, 124). It was used to relieve clinical symptoms in two patients with LPG who had failed to respond to statins (125). However, there was still a lack of more widespread attempts.

The other drugs that are still in clinical use today are statins. As a potent inhibitor of 'β-hydroxy β-methylglutaryl-CoA' ('HMG-CoA') reductase, statins can increase the expression of the LDL receptor in the hepatocytes' cell membranes. In addition, statins also have multiple effects, such as reducing TG, improving endothelial function, and anti-oxidation (126). Statins can reduce patients' blood lipid levels but can also effectively lower urinary protein levels and retain renal function. There were very few cases in which statin therapy alone was evaluated (16, 90, 94, 108, 111, 119, 125, 127).

Renin-angiotensin-aldosterone system inhibitor (RAASi) therapy shows reno-protective effects in various forms of proteinuric CKD. Therefore, although there is no evidence that ACEIs and ARBs are effective in LPG, their administration can be considered suitable both for blood pressure control and to reduce proteinuria among LPG patients. Some case reports have suggested that antilipidemic drugs combined with ACEIs and ARBs are an effective treatment for LPG (128). Recently, SGLT2 inhibitors proved to be beneficial for patients who have proteinuric CKD with or without diabetes. With evidence that SGLT2 inhibitors might be safe and useful in hereditary renal diseases such as Alport syndrome (129), whether SGLT2 inhibitors could be a potential treatment for LPG remains to be further investigated.

### Plasma Adsorption Therapy

Adsorption therapy also showed a certain effect. LDL apheresis was used when the patient had a poor response to traditional drug therapy. However, allergic reactions to LDL-apheresis are a concern (90). In 2009, a heparin-induced extracorporeal lipoprotein precipitation (HELP) system was first used to treat a 60-year-old white woman with proteinuria, high blood pressure, and renal failure (90). The basic principle of this system is to acidify plasma pH to ~5 *in vitro* and then use heparin to form a polymer with LDL-cholesterol, LP (a), fibrinogen, and triglyceride to precipitate them out (130, 131). After 25 courses of treatment, the patient achieved significant clinical improvements, including decreased urinary protein, blood creatinine and lipid levels.

Protein A, immunosorbent may also be a treatment option. Since protein A has a strong affinity for the FcγR of IgG, this effect can compensate for the lack of an FcRγ. This technique has previously been shown to reduce proteinuria levels in patients with a variety of nephropathies, including diabetic nephropathy, IgA nephropathy, and amyloidosis (132). In a small pilot study involving 13 cases, immunoadsorption was administered for 10 cycles per session and 10 sessions as a course. A total of 30 L of plasma was regenerated in each course. Apart from proteinuria reduction, a repeated renal biopsy (*n* = 12) showed that intraglomerular lipoprotein thrombi almost disappeared. The proteinuria of six patients returned to baseline levels within 12 months. Four recurrent patients received repeat immunoadsorption treatment and showed repeated efficacy (97). The strategy is advantageous in being rapidly therapeutic and having relatively stable effects. However, disadvantages include invasive procedure complications, high cost and risk of infection.

### Other Possible Treatments

Other lipid-lowering drugs, such as niacin (133) and apoC-III monoclonal antibodies, as well as non-pharmaceutical approaches, such as lipoprotein apheresis, may also be promising (134, 135). Other antioxidants, apart from probucol, including vitamin C, polyphenols, N-acetylcysteine, allopurinol, natural polysaccharides, pentoxifyllin, and bardoxolone methyl, may be candidates to study antioxidant efficacy, since antioxidant therapy apparently reduces CKD progression (136, 137). However, high interindividual variability and off-target effects, such as body weight reduction, need to be further investigated. The anticonvulsant topiramate, which induces weight loss and a moderate reduction in plasma lipids and glucose, has recently been reported to protect *APOE*-deficient mice from kidney damage. Thus, it could be investigated in drug repurposing studies for the treatment of glomerular lipidosis (138).

Gene-based therapeutics, pioneered for the treatment of monogenic inherited retinal disease, are being actively investigated as new treatments for acquired retinal disease. Gene therapy could also be tried in the future, i.e., by CRISPR/Cas9 technology, since it is widely believed that LPG is an inherited disease caused by an abnormality in the *APOE* gene (2, 9).

## Conclusions

Current studies have found that several mutations of the *APOE* gene can lead to LPG, and most of these mutations are concentrated in some hot spots. Future functional research on LPG focusing on hot spot regions for gene mutations may be helpful to explore the specific pathogenesis of the disease and to develop target drugs. It is worth exploring whether different genotypes lead to differences in clinical phenotypes after increasing the number of patients who are enrolled and who can be stratified.

Although the symptoms of LPG are common and fixed, some cases still cannot be explained by the current theory. For example, in 2015, a patient presented with a large number of macrophage infiltrates in the glomerulus. In most cases, macrophage infiltration is not present in the glomerular capillaries of patients with LPG (91). In 2018, a new variant of the *APOE* gene (*APOE* Toyonaka) was discovered. Patients with this gene mutation presented with kidney pathology that resembled membranous nephropathy (MN) rather than LPG (139, 140). For cases such as this, it is still not clear if they belong to a new disease category or a different spectrum of LPG (27, 28, 141). *APOE*-related disorders were recently summarized, mainly including *APOE*2 homozygote glomerulopathy (HLP), MN-like *APOE* disease, and LPG (141). All the cases above indicate that the incidence and manifestations of LPG cannot be fully explained by existing theories. In addition to the new theory of FcRγ pathogenesis, other causes of LPG may be discovered in the future.

The current clinical diagnosis and treatment of LPG still needs to be improved. As the clinical manifestations of LPG are often non-specific, the actual incidence of LPG is underestimated. The diagnosis is beyond the scope of primary care hospitals, and conventional nephropathy treatment has proven ineffective, so the sensitivity of the diagnosis needs to be increased so that more patients can be treated properly. In terms of treatment, effective drugs are not commonly used in practice, and therapy has not been commonly used, so new drug research and development are needed. For those who have been diagnosed, indicators for clinical surveillance are still lacking. As a rare disease, an international registry may be worthwhile, as registries are essential for epidemiological data and collaborative research.

## Author Contributions

M-sL: collected data, conceived, and wrote the manuscript. YLi: collected data and wrote the manuscript. YLiu: collected data. X-jZ: revised the manuscript critically for important intellectual content and supervised the research group and has given the final approval of the version to be published. HZ: edited the manuscript and has given the final approval of the version to be published. All authors contributed to the article and approved the submitted version.

## Funding

Support was provided by National Science Foundation of China (82022010, 82131430172, 81970613, 82070733, 82000680, and 82070731); Academy of Medical Sciences—Newton Advanced Fellowship (NAFR13_1033); Beijing Natural Science Foundation (Z190023); Fok Ying Tung Education Foundation (171030); and CAMS Innovation Fund for Medical Sciences (2019-I2M-5-046). The funders had no role in study design, data collection and analysis, decision to publish, or preparation of the manuscript.

## Conflict of Interest

The authors declare that the research was conducted in the absence of any commercial or financial relationships that could be construed as a potential conflict of interest.

## Publisher's Note

All claims expressed in this article are solely those of the authors and do not necessarily represent those of their affiliated organizations, or those of the publisher, the editors and the reviewers. Any product that may be evaluated in this article, or claim that may be made by its manufacturer, is not guaranteed or endorsed by the publisher.

## Abbreviations

LPG: Lipoprotein glomerulopathy
LCAT: Lecithin-cholesterol acyltransferase
LDLR: Low-density lipoprotein receptor
HSPG: Heparan sulfate proteoglycan
HLP: Hyperlipoproteinemia
LRP: LDL receptor-associated protein
GVHD: Graft-versus-host disease
FcRγ: Fc receptor gamma chain
CML: carboxymethyllysine
HNE: hydroxynonenal
MDA: Malondialdehyde
aHUS: Atypical hemolytic uremic syndrome
PPAR: Peroxisomal proliferator-activated receptor
LPL: Lipoprotein lipase
HMG-CoA: β-hydroxy-β-methylglutaryl-CoA
RAASi: Renin-angiotensin-aldosterone system inhibitor
HELP: Heparin-induced extracorporeal lipoprotein precipitation
MN: membranous nephropathy
IDL: Intermediate density lipoprotein
VLDL: Very low-density lipoprotein
HDL: High-density lipoprotein
TC: Total cholesterol
TG: Total triglyceride.
