# Supplementary material for: An Updated Review and Meta Analysis of Lipoprotein Glomerulopathy
Source: Front Med (Lausanne). 2022 May 6;9:905007. doi: 10.3389/fmed.2022.905007 (PMC9120586; doi:10.3389/fmed.2022.905007)
Supplement: Supplementary file 2 [file Table_2.DOCX]

**Additional file 2: Clinical manifestations of LPG patients in literature review.**

| Number of cases | Blood pressure (mmHg) | Albumin (g/L) | Serum creatinine (μmol/L) | BUN (mmol/L) | eGFR  (ml/min/1.73m^2^) | APOE (mg/dL) | TC (mmol/L) | TG (mmol/L) | LDL (mmol/L) | HDL (mmol/L) | URBC (HPF) | 24h UPRO (g) | Ref |
| --- | --- | --- | --- | --- | --- | --- | --- | --- | --- | --- | --- | --- | --- |
| 8 |  | 25.2±6.56 |  |  | 83.6±21.6 | 4.8±1.4 | 6.2±2.2 | 2.8±0.94 |  |  |  | 6±3.5 | (1) |
| 1 | 207/138 | 27 | 184.8 |  | 37 |  | 13 | 0.9 | 10.6 | 1.9 | 0 | 11.7 | (2) |
| 1 | 115/77 | 38 | 45.8 | 4.2 | 145 |  | 8.5 | 1.6 | 6.4 | 1.3 | 6.5 | 4 | (3) |
| 2 |  | 38.1 | 108.1 |  |  | 7.4 | 5.2 | 2.29 | 3.34 | 1.2 |  |  | (4) |
| 6 |  |  |  |  |  | 8.3±2.7 | 7.7 | 5.1 | 6.1±0.5 |  |  |  | (5) |
| 2 | 153/99 | 37.4 | 67.2 | 5.7 | 93.1 | 4.4 | 5.6 | 2.2 | 2.8 | 1.7 | 11 | 3.8 | (6) |
| 35 | 115 | 26.4 | 98.1 |  | 79 | 9.3 | 7 | 3.5 | 4.3 | 1.5 |  |  | (7) |
| 1 |  | 29.6 |  |  |  | 5.6 | 4 | 1.7 | 1.7 | 1.7 |  | 4.4 | (8) |
| 1 | 220/160 | 26.6 | 282.9 | 18.5 | 20.1 |  | 7.1 | 2.9 |  |  |  | 7.9 | (9) |
| 2 |  |  |  |  |  | 10.6 |  |  |  |  |  |  | (10) |
| 7 |  |  | 75.6±16 |  | 103 | 20.9±15.2 | 6±0.9 | 2.6±1.1 | 3.3±1 | 1.1±0.2 |  | 3.4±0.4 | (11) |
| 1 | 148/90 | 23 | 72 |  | 115.2 |  | 9.3 | 4.7 | 1.2 | 1.1 |  | 5.3 | (12) |
| 13 |  | 29.8 | 99.9 |  |  | 9.8 | 6.4 | 2.7 | 3.1 | 1.4 |  | 4±3.1 | (13) |
| 16 | 136/87 | 32.6 | 102 | 7.8 |  | 13.4 | 5.7 | 3.7 | 2.4 | 1.2 |  | 3.8 | (14) |
| 4 |  |  | 676 |  | 5.3 | 7.5 | 5.5 | 1.3 | 2.7 | 1.5 |  |  | (15) |
| 13 |  |  |  |  |  | 14.8±5.9 | 5.7±0.7 | 3.6±1 | 2.5±0.2 | 1.2±0.5 |  | 3.6±1 | (16) |
| 8 | 136/90 | 32.6±5.5 | 99.6±36.6 | 7.6±4.6 | 91.7 | 19.3±25.5 | 5.9±1.5 | 4.4±1.8 | 2.9±0.9 | 1.3±0.5 |  | 4.5±3.9 | (17) |
| 3 | 150/100 | 23.3 | 139.9 | 10.4 | 58.4 |  | 8.1 | 3.7 | 4.6 | 1.4 | 5 | 1.2 | (18) |
| 2 | 122/79 | 24 | 105.9 | 10.3 | 63.6 |  | 7.5 | 3.3 |  |  | 8 | 6.2 | (19) |
| 1 | 140/88 |  | 53 |  | 127 | 11.1 | 5.7 | 4 |  |  |  | 5 | (20) |
| 2 |  | 24.5 | 92.8 | 6.4 | 72.7 |  | 8 | 2 | 5.1 | 1.5 |  | 8 | (21) |
| 1 | 164/114 |  | 229.8 |  | 32.1 | 14.6 | 12.9 | 5.5 |  |  |  | 3.9 | (22) |
| 1 |  | 27.3 | 139 | 10.3 | 46.7 |  | 6.6 | 1.7 | 4.6 | 1.3 | 8 | 5.1 | (23) |
| 3 | 125/80 | 26.8 | 83.5 |  | 101.6 |  | 6.3 | 2.6 |  |  |  | 4.1 | (24) |
| 1 |  | 29 | 61 | 5.7 | 120.5 |  |  |  |  |  |  | 5.9 | (25) |
| 1 |  |  | 42 |  | 172.6 | 11 | 4.2 | 1.4 | 2.1 | 1.3 |  | 3.7 | (26) |
| 1 | 151/91 | 30.3 | 85.6 |  | 98 |  | 4.4 | 1.7 | 2.4 | 1.1 | 16 | 5.2 | (27) |
| 1 |  | 35 | 1068 |  | 5.2 | 38 | 4.8 | 0.9 |  |  |  |  | (28) |
| 1 | 125/81 | 23.7 | 118 | 6.2 | 69.7 | 8.3 | 8.3 | 2.7 | 3.9 | 2 |  | 2.9 | (29) |
| 1 | 140/90 |  | 77.5 | 5.3 | 103.7 | 7.4 | 5 | 3 | 3 | 0.8 |  | 2.2 | (30) |
| 2 | 140/90 | 38.8 | 78.3 | 3.9 | 95.2 |  | 6 | 7 | 1.3 | 3.3 |  | 2.9 | (31) |
| 1 | 120/77 | 34.8 | 45 | 4.1 | 150 |  | 10.1 | 5.1 | 7.1 | 1.2 |  | 1.1 | (32) |
| 1 | 140/80 |  |  |  |  |  | 5.9 | 2.3 |  |  |  | 2.6 | (33) |
| 1 | 130/60 | 32 |  |  |  |  | 8 | 1.9 | 5.4 | 2.3 |  | 3.6 | (34) |
| 1 | 160/100 | 30.2 | 91.1 | 5 | 72.6 |  | 6.7 | 2.8 | 4.1 |  |  | 2.4 | (35) |
| 1 |  | 32.7 | 96 |  | 95.2 |  | 3.7 | 1.4 | 1.5 | 1.6 |  | 1.4 | (36) |
| 2 | 145/90 |  |  |  |  |  |  |  |  |  |  |  | (37) |
| 10 | 140/85 | 26.2±5.1 | 100.7±19.5 |  | 83 |  | 8.2±2.5 | 4.5±1.6 | 4.4±1.1 | 1.5±0.2 | 21.2±18.9 | 5.1±1.8 | (38) |
| 2 | 145/90 |  | 90.8 |  | 55.4 | 5.6 | 4.9 | 2.3 | 2.8 | 1.3 |  | 2 | (39) |
| 1 | 124/70 | 22.5 | 121.7 | 13.2 | 68.6 | 8.5 |  |  | 5.7 | 1.3 |  | 5.9 | (40) |
| 1 |  | 28.4 |  | 4 |  |  | 7.3 | 4 | 4.2 | 1.5 |  |  | (41) |
| 1 | 110/90 | 23.6 | 50 | 6.8 | 154.2 | 25.3 | 6.2 | 4.5 | 3.5 | 1.4 |  | 3.9 | (42) |
| 1 |  |  | 127.4 | 5.7 | 55.8 |  | 4.8 | 3.2 | 3.1 | 1 | 1 |  | (43) |
| 1 | 100/80 | 39 |  | 10.3 |  |  | 5.7 | 3.7 | 4.5 | 0.9 |  |  | (44) |
| 3 |  | 24 | 59 | 4 | 116.3 | 11.2 | 6.9 | 4.1 | 5.1 | 1.7 | 8.5 | 13.6 | (45) |
| 1 | 150/80 | 19 | 88 | 5.4 | 76.4 | 12 | 10 | 2 | 7 |  |  | 5.4 | (46) |
| 1 | 180/102 | 28.1 | 132.6 |  | 51.4 |  | 6.2 | 4 |  | 2.3 |  | 3.6 | (47) |
| 3 |  | 31.7 | 84 | 6.2 | 94.6 | 18.3 | 6.7 | 7.9 |  | 1.6 |  | 6.3 | (48) |
| 2 | 145/95 | 26.7 | 108.9 | 6.8 | 68.8 | 9.6 | 7.4 | 3.3 | 3.8 | 1.3 | 20 | 3.9 | (49) |
| 2 | 137/100 |  | 91.5 |  | 80.11 | 10.5 | 7.1 | 2.4 | 4.1 | 1 | 10.5 | 5.1 | (50) |
| 2 | 165/97 | 26.5 | 155 | 5.6 | 55 |  | 7.6 | 2.2 | 4.3 | 1.8 | 10.8 | 7.4 | (51) |
| 1 | 160/105 | 37.4 | 100 | 3.8 | 82 | 6.3 | 3.8 | 4.1 | 1.3 | 0.8 |  | 3.4 | (52) |
| 3 | 150/100 | 23.3 | 139.9 | 10.4 | 62.8 |  | 8.1 | 3.7 | 4.6 | 1.4 | 5 | 1.2 | (53) |
| 1 | 120/75 | 33.6 | 89 | 7.7 | 94.4 |  | 14.4 | 1.5 |  | 1.4 |  |  | (54) |
| 4 |  |  |  |  |  | 9.3 | 6.1 | 3.6 | 2.4 | 1.5 |  |  | (55) |
| 1 | 165/98 |  | 149 | 23.6 | 42.3 |  | 7.3 | 3.2 | 4.7 | 2.1 |  | 3.7 | (56) |
| 1 | 135/75 |  | 86 | 3.3 | 77.2 |  | 8.2 | 1.9 | 5.6 | 2.2 |  |  | (57) |
| 1 | 128/85 | 25.5 | 145.4 |  | 48 |  | 5.3 | 1.9 |  |  |  | 2.8 | (58) |
| 3 |  | 34.5 | 67.5 |  | 109.6 | 8.7 | 5.2 | 1.7 |  |  |  | 2.2 | (59) |
| 2 | 151/86 | 29.2 | 138.1 | 9.3 | 54 |  | 7.9 | 2.2 | 4.1 |  |  | 2.4 | (60) |
| 1 | 145/100 | 23.6 | 37 | 13.3 | 157.7 | 16.6 | 13.1 | 6.1 |  |  |  | 8.7 | (61) |
| 1 | 170/100 | 22 | 116 | 12.1 | 76.2 |  | 10.1 | 2.1 | 2.8 | 1.2 | 4.5 | 6.5 | (62) |
| 1 | 140/100 | 33.2 | 91 |  | 73.6 | 14 | 12.1 | 20.6 | 4.5 |  | 1 | 4 | (63) |
| 1 |  | 19.5 | 300 |  | 26.2 | 10.5 |  |  |  |  |  | 4.6 | (64) |
| 1 | 130/90 | 28.1 |  |  |  |  | 6.28 |  |  |  |  |  | (65) |
| 1 | 120/75 | 39 | 65 | 4.4 | 141.5 |  | 6.3 | 5.6 | 2.6 | 1.2 | 5 | 0.6 | (66) |
| 1 | 140/95 | 20 | 71 | 10.7 | 96.1 |  | 5.8 | 3.7 | 3 | 1.16 |  | 5.6 | (67) |
| 1 | 120/80 | 21 | 58 | 6.7 | 138 |  | 5 | 2.2 | 2.2 |  |  | 2.1 | (68) |
| 1 | 110/70 | 25 |  |  |  | 13.7 | 5.4 | 4.6 |  |  |  | 4 | (69) |
| 1 | 128/68 | 37 | 71.6 |  | 85 | 18.4 | 7.9 | 2.5 | 5 | 1 |  | 3.1 | (70) |
| 1 | 137/94 | 36 | 70.7 | 3.9 | 85.6 | 9.2 | 6.4 | 4.7 | 2.3 | 0.9 |  | 4.1 | (71) |
| 1 | 110/72 | 31 | 106.1 |  | 50.8 | 7.6 | 5.9 | 2.9 | 2.9 |  |  | 5.1 | (72) |
| 1 | 170/120 | 16 | 120.2 |  | 71.8 | 14.6 | 22.9 | 6.2 |  | 1.2 | 24.5 |  | (73) |
| 1 | 128/70 | 42 | 59.2 | 2.9 | 132.6 | 3.1 | 4.2 | 3 | 1.5 |  |  | 0.5 | (74) |
| 1 |  |  | 85.8 |  | 93.9 | 5.2 | 2.3 | 2.4 | 3.5 | 1 | 3 |  | (75) |
| 1 | 117/59 | 41 | 50 | 3 | 66.1 | 11.6 | 6.8 | 1.7 |  |  | 7.5 | 0.9 | (76) |
| 1 | 132/70 | 43 | 46.9 | 5.3 | 86.9 |  | 3.3 | 1.6 |  |  |  | 0.8 | (77) |
| 14 |  |  | 123.8±8.8 |  | 75.9±17.3 | 16.8±6.5 | 7.6±3.2 | 4.1±2.3 |  |  |  | 4.6±7.2 | (78) |
| 1 |  | 21 | 61.9 | 5.9 | 104 | 6.9 | 5.4 | 1.5 |  | 1.1 |  | 24 | (79) |
| 2 | 125/75 |  |  |  | 77 | 25.3 | 5.3 | 2 | 2.5 | 1 | 0 | 2 | (20) |
| 1 | 140/88 | 34 | 36 | 4.3 | 163.7 | 19.9 | 6.9 | 5.7 |  | 0.6 | 15 | 1.5 | (80) |
| 1 | 150/70 | 31 | 123.8 | 6.9 | 56.9 | 8.2 | 4.4 | 2.4 | 1.6 | 0.6 | 6.5 | 1.5 | (81) |
| 1 | 128/76 | 43 | 88 | 7.8 | 83.8 | 9.2 | 4.8 | 1 | 2.3 | 1.6 |  | 1.3 | (82) |
| 1 | 160/85 | 38 | 123.8 | 2.7 | 134 | 29.6 | 6.3 | 5.5 |  |  |  | 2 | (83) |
| 1 | 169/90 | 41 |  | 5.5 | 78 | 38.8 | 18.6 | 11.5 |  |  |  | 2.2 | (84) |
| 1 | 140/92 | 44 | 88.4 | 3.9 | 97 | 11.2 | 4.7 | 1.6 | 2.2 | 8.8 |  | 1.9 | (85) |
| 1 |  |  | 424.3 | 25.6 | 16 | 16.8 | 5.3 | 1.7 |  |  |  |  | (86) |
| 1 |  |  |  |  | 81.6 | 14 | 7.1 | 2.2 |  |  | 12 | 9 | (87) |
| 1 | 144/88 |  | 79.6 |  | 77 | 14 | 6.2 | 1.6 |  |  |  | 1.6 | (88) |
| 5 |  |  |  |  |  | 13.9 | 6.2 | 3.1 | 2.6 | 1 |  |  | (89) |
| 1 | 160/85 | 12 | 168 | 17.4 | 57 | 29.6 | 13.8 | 5.5 |  |  |  | 5 | (90) |
| 1 | 120/100 | 34 | 29.2 | 4 | 170.7 | 19.9 | 6 | 3.1 |  |  | 5.5 |  | (91) |
| 1 | 104/70 | 42 | 21.2 | 4.3 | 157 | 12.2 | 4.9 | 1.5 |  |  | 2.5 |  | (92) |
| 1 | 162/88 | 19 | 88 |  | 59.5 | 12.1 | 17.5 | 3.9 | 3.1 | 28 |  | 4.4 | (93) |
| 4 | 140/92 |  |  |  |  |  | 9.8 | 5.9 |  |  |  | 1.5 | (94) |
| 1 | 140/80 |  |  |  |  | 19.9 |  |  |  | 0.6 |  |  | (95) |
| 1 |  |  | 88.4 |  | 87.4 |  | 6.3 | 2.3 | 4.3 | 1 |  | 7 | (96) |
| 1 |  | 17 | 203.3 |  | 36.5 |  | 5.7 | 2.5 | 3.7 | 0.9 |  | 9.8 | (97) |
| 1 |  | 47 | 115 |  | 70.2 |  |  | 2.5 | 3 | 0.9 |  | 12 | (98) |
| 1 | 140/90 |  | 114.9 |  | 44 |  | 4.6 | 1 | 3.3 | 1 |  | 3.2 | (99) |
| 1 |  | 26 | 61.9 |  | 144.7 |  | 13.8 | 7.2 |  |  |  | 10.7 | (100) |
| 2 |  | 27 | 514.5 |  | 53.7 | 7.2 | 5.7 | 5.9 | 3.6 | 0.9 | 54 | 7.6 | (101) |
| 1 | 156/95 | 40.2 | 82.2 |  | 69.2 |  | 10.1 | 3.9 | 7 | 1.6 |  |  | (102) |
| 2 |  | 27 | 121.6 |  | 75.2 | 8 | 6.4 | 2.3 | 3.5 | 0.9 |  | 3.7 | (103) |
| 1 | 160/100 |  | 79.6 |  | 63 |  | 9.6 | 2.7 | 6.9 |  |  | 5 | (104) |
| 1 | 177/115 | 29 | 168 | 10.4 | 57 |  | 2.4 | 2.7 | 3.4 | 1.6 |  | 9.7 | (78) |
| 2 | 145/95 | 41 | 115 | 9 | 90 |  | 7.7 | 3.5 |  |  |  | 1.9 | (105) |
| 1 |  | 30 |  |  | 55 |  | 6.5 | 3.6 |  | 0.8 |  | 1.3 | (106) |
| 1 |  | 31.6 | 86.6 |  | 92.5 | 2 | 5.4 | 5.4 | 3 | 1.3 |  | 8.8 | (107) |
| 1 | 171/106 | 27 | 907 | 31.7 | 6.6 |  | 9.5 | 1.6 | 7.6 | 1.5 |  |  | (108) |
| 1 | 268/168 | 31 | 1859 | 39.9 | 2.8 |  | 3.6 | 0.7 |  |  |  | 6.4 | (109) |
| 1 |  |  | 70 |  | 143 |  | 7.8 | 1.1 |  |  |  | 8 | (110) |
| 1 | 190/130 | 17 | 134 |  | 32.3 |  |  |  |  |  |  | 12.6 | (111) |

1. Yang M, Weng Q, Pan X, Hussain HMJ, Yu S, Xu J, Yu X, Liu Y, Jin Y, Zhang C, et al. Clinical and genetic analysis of lipoprotein glomerulopathy patients caused by APOE mutations. *Mol Genet genomic Med* (2020) **8**:e1281. doi: 10.1002/mgg3.1281

2. Lui DTW, Lee ACH, Yap DYH, Chan GSW, Tan KCB. A young Chinese man with nephrotic syndrome due to lipoprotein glomerulopathy. *J Clin Lipidol* (2019) **13**:251–253. doi: 10.1016/j.jacl.2018.12.004

3. Ku M, Tao C, Zhou A-A, Cheng Y, Wan Q-J. A novel apolipoprotein E mutation (p.Arg150Cys) in a Chinese patient with lipoprotein glomerulopathy. *Chin Med J (Engl)* (2019) **132**:237–239. doi: 10.1097/CM9.0000000000000050

4. Xie W, Xie Y, Lin Z, Xu X, Zhang Y. A novel apolipoprotein E mutation caused by a five amino acid deletion in a Chinese family with lipoprotein glomerulopathy: a case report. *Diagn Pathol* (2019) **14**:41. doi: 10.1186/s13000-019-0820-6

5. Zou GM, Zhuo L, Tan M, Li WG. [Clinicopathologic features of lipoprotein glomerulopathy: observation of 6 cases]. *Chin Mde J* (2018) **98**:2910–2913. doi: 10.3760/cma.j.issn.0376-2491.2018.36.007

6. Wu H, Yang Y, Hu Z. The Novel Apolipoprotein E Mutation ApoE Chengdu (c.518T＞C, p.L173P) in a Chinese Patient with Lipoprotein Glomerulopathy. *J Atheroscler Thromb* (2018) **25**:733–740. doi: 10.5551/jat.41996

7. Hu Z, Huang S, Wu Y, Liu Y, Liu X, Su D, Tao Y, Fu P, Zhang X, Peng Z, et al. Hereditary features, treatment, and prognosis of the lipoprotein glomerulopathy in patients with the APOE Kyoto mutation. *Kidney Int* (2014) **85**:416–424. doi: 10.1038/ki.2013.335

8. Li W, Wang Y, Han Z, Luo C, Zhang C, Xiong J. Apolipoprotein e mutation and double filtration plasmapheresis therapy on a new Chinese patient with lipoprotein glomerulopathy. *Kidney Blood Press Res* (2014) **39**:330–339. doi: 10.1159/000355810

9. Wu Y, Chen X, Yang Y, Wang B, Liu X, Tao Y, Fu P, Hu Z. A case of lipoprotein glomerulopathy with thrombotic microangiopathy due to malignant hypertension. *BMC Nephrol* (2013) **14**:53. doi: 10.1186/1471-2369-14-53

10. 韩鸿玲, 林珊, 孙丽莎, 张鹏, 翟德佩. 一个脂蛋白肾小球病家系中apoE基因的九个碱基缺失. 中华医学遗传学杂志 (2012) **29**:141–144.

11. Han J, Pan Y, Chen Y, Li X, Xing G, Shi J, Hou P, Zhang H, Wang H. Common apolipoprotein e gene mutations contribute to lipoprotein glomerulopathy in China. *Nephron Clin Pract* (2010) **114**:260–267. doi: 10.1159/000276578

12. Cheung CY, Chan AOK, Chan YH, Lee KC, Chan GPT, Lau GTC, Shek CC, Chau KF, Li CS. A rare cause of nephrotic syndrome: lipoprotein glomerulopathy. *Hong Kong Med J* (2009) **15**:57–60.

13. Xin Z, Zhihong L, Shijun L, Jinfeng Z, Huiping C, Caihong Z, Daxi J, Leishi L. Successful treatment of patients with lipoprotein glomerulopathy by protein A immunoadsorption: a pilot study. *Nephrol Dial Transplant* (2009) **24**:864–869. doi: 10.1093/ndt/gfn555

14. Zhang B, Liu ZH, Zeng CH, Zheng JM, Chen HP, Li LS. Clinicopathological and genetic characteristics in Chinese patients with lipoprotein glomerulopathy. *J Nephrol* (2008) **21**:110–117.

15. Luo B, Huang F, Liu Q, Li X, Chen W, Zhou S-F, Yu X. Identification of apolipoprotein E Guangzhou (arginine 150 proline), a new variant associated with lipoprotein glomerulopathy. *Am J Nephrol* (2008) **28**:347–353. doi: 10.1159/000111828

16. Zhang B, Liu Z, Zeng C, Zheng J, Chen H, Zhou H, Li L. Plasma level and genetic variation of apolipoprotein E in patients with lipoprotein glomerulopathy. *Chin Med J (Engl)* (2005) **118**:555–560.

17. Chen H, Liu Z, Gong R, Tang Z, Zeng C, Zhu M, Wang J, Zhou H, Li L. Lipoprotein glomerulopathy: clinical features and pathological characteristics in Chinese. *Chin Med J (Engl)* (2004) **117**:1513–1517.

18. Hu Z, Zhang X, Sha Z, Yang L, Huang S. [Clinical and pathological analyses of lipoprotein glomerulopathy]. *J Sichuan Univ Med Sci Ed* (2004) **35**:442–444.

19. Chang C-F, Lin C-C, Chen J-Y, Yang A-H, Shiao M-S, Kao J-T, Yang W-C. Lipoprotein glomerulopathy associated with psoriasis vulgaris: report of 2 cases with apolipoprotein E3/3. *Am J kidney Dis* (2003) **42**:E18-23. doi: 10.1016/s0272-6386(03)00798-4

20. Ieiri N, Hotta O, Taguma Y. Resolution of typical lipoprotein glomerulopathy by intensive lipid-lowering therapy. *Am J kidney Dis* (2003) **41**:244–249. doi: 10.1053/ajkd.2003.50016

21. Yang AH, Ng YY, Tarng DC, Chen JY, Shiao MS, Kao JT. Association of apolipoprotein E polymorphism with lipoprotein glomerulopathy. Report of 2 cases with a new genotype and comparison of the relative frequencies of apolipoprotein E isoforms in lipoprotein glomerulopathy and in the general population. *Nephron* (1998) **78**:266–270. doi: 10.1159/000044933

22. Zhang P, Matalon R, Kaplan L, Kumar A, Gallo G. Lipoprotein glomerulopathy: first report in a Chinese male. *Am J kidney Dis* (1994) **24**:942–950. doi: 10.1016/s0272-6386(12)81066-3

23. 鲍清辉, 黄新艳. 中医论治脂蛋白肾病1例. 中国中医药现代远程教育 **19**:3.

24. 何海硕, 朱圣红, 崔慧单, 赵雪红, 李杏利. 双重血浆滤过治疗家族性脂蛋白肾病的护理. 基层医学论坛 (2020) **024**:1686–1687.

25. 雷蕾, 刘承玄, 张鹏程, 熊维建. 脂蛋白肾病1例报道及文献复习. *中国现代医生 2020年58卷25期 150-154,158页* (2020)

26. 邱莲, 熊加川. 苯扎贝特治疗儿童ApoE Tokyo(Arg25Cys)突变型脂蛋白肾病1例. 临床肾脏病杂志 (2020) **v.20**:89–92.

27. 杨智, 吴鸿雁, 胡章学. APOE Osaka/Kurashiki变异致脂蛋白肾病藏族患者一例. 中华医学遗传学杂志 (2020) **37**:166–169.

28. 张锦, 陈徐涛, 黄刚, 邱江, 陈国栋, 陈立中, 费继光, 王长希. 移植肾脂蛋白肾病的临床研究. 中华器官移植杂志 (2019) **40**:620–623.

29. 黄琼, 曾庆明, 易无庸, 郑义侯, 熊吉龙, 邵牧民. 脂蛋白肾小球病1例报告并文献复习. 南昌大学学报(医学版) (2015) **055**:100–104.

30. 薛汝群, 陈万佳, 钟逸斐, 赵仲华, 蔡小凡, 刘学光. 中药治疗1例脂蛋白肾病. 复旦学报(医学版) (2017) **44**:392–394.

31. 董晓明, 杨帆, 吴淋淋, 罗萍. 脂蛋白肾病临床病理特点及文献回顾性分析. 中国中西医结合肾病杂志 (2016) **17**:805–807.

32. 贾莉敏, 张建江, 史佩佩, 曾慧勤, 窦文杰, 曾丽娜, 贾东华, 陈莹华, 孔祥东. 儿童脂蛋白肾病1例报告并文献复习. 临床儿科杂志 (2016)

33. 尹自长, 岳书玲, 江启锋, 张欢, 袁小六, 耿建. 脂蛋白肾小球病合并IgA肾病一例病理诊断. 临床肾脏病杂志 (2015) **15**:762–764.

34. 罗春明, 黄向阳. 误诊为肾病综合征的成人脂蛋白肾病一例并文献复习. 医学临床研究 (2015) **000**:623–625.

35. 钟春梅, 熊良伟. 脂蛋白肾病1例报道. 当代医学 (2014)12–13.

36. 吴瑞娟, 曾芳, 韩勇, 马林, 陈东生. 1例脂蛋白肾病患者的3种治疗方案分析及药学监护. 中国药师 (2014)1927–1928.

37. 晋中恒, 郭术莲, 蒋松, 王建华. 脂蛋白肾病治验2例. 临床合理用药杂志 (2013)138–139.

38. 陈亭宇. 10例脂蛋白肾病临床表现及病理特征. 中国保健营养旬刊 (2013)595–596.

39. 张祥文, 李冰心, 贾中尉, 李丽华, 何川鄂. 家族性脂蛋白肾病8例临床分析. 广东医学 (2012) **033**:349–350.

40. 胡华, 张新民. 脂蛋白肾病 1 例报告及文献复习. 中南大学学报 (医学版) (2012) **37**:320–324.

41. 张磊, 许冬梅, 张爱平, 王艳侠. 脂蛋白肾病1例报告并文献复习. 实用医药杂志 (2011) **28**:900–901.

42. 杨海峰, 王智园, 黄清明, 罗碧怡, 李楚天, 何青莲. 脂蛋白肾小球病1例及文献复习. 中国中西医结合肾病杂志 (2009)

43. 易韦, 文安智. 脂蛋白肾病 1 例. 贵阳医学院学报 (2009) **34**:479.

44. 李龙海, 董葆, 王建, 李秀勇, 吴振球, 李庆士, 李振军, 孙静静, 姜传学, 邹万忠. 脂蛋白肾病1例报告及文献复习. 安徽医学 (2009) **30**:581–582.

45. 徐利芬, 陈佳, 黄凇崧, 孟青, 曾晓. 脂蛋白肾小球病的临床病理分析. 中华病理学杂志 (2008) **37**:408–409.

46. 黎伟, 王成玉, 廖蕴华, 冯震博. 脂蛋白肾病1例报告及文献复习. 广西医学 (2007) **029**:591-592,封3.

47. 李梦, 彭红英. 脂蛋白肾病1例个案报道. 医药前沿 (2018) **008**:172–173.

48. 孙丽莎, 王杨, 李昕, 韩鸿玲, 翟德佩. 载脂蛋白E的检测对诊断脂蛋白肾病的临床意义. 检验医学 (2006) **021**:187–188.

49. 吕永曼, 曾红兵, 徐钢, 韩敏, 何晓峰, 官阳, 武忠弼. 脂蛋白肾小球病二例. 中华病理学杂志 (2006) **35**:440–441.

50. 潘永利, 陈育青, 张宏, 李侠, 邢广群, 师军华, 侯平, 王海燕. 四例脂蛋白肾病患者载脂蛋白E基因突变筛查. 中华肾脏病杂志 (2006) **022**:449–453.

51. 张静，刘彦仿，刘健. 脂蛋白肾病2例临床病理分析. 诊断病理学杂志 (2005) **12**:188–191.

52. 陈燕, 王庆文. 全血脂蛋白吸附治疗脂蛋白肾病. 肾脏病与透析肾移植杂志 (2004) **13**:493.

53. 胡章学, 张秀辉, 沙朝晖, 杨立川, 黄颂敏. 脂蛋白肾病临床与病理分析. 四川大学学报 医学版 (2004) **35**:442–444.

54. 梁兰青, 阿孜古丽, 冯维, 邹赛英. 脂蛋白肾小球病——附1例报告及文献复习. 西北国防医学杂志 (2002)

55. 龚如军，刘志红，陈朝红. 载脂蛋白E及其基因变异在脂蛋白肾病发病中的作用. 肾脏病与透析肾移植杂志 (2001) **10**:329–334.

56. 姚坚坚. 脂蛋白肾病1例. 实用医学杂志 (2000) **09**:762.

57. 姜傥, 许乃贵, 邹万忠, 黄锋先, 关伟明, 吴义方, 李鵕, 董秀清. 脂蛋白肾病——一种新型的肾小球疾病伴进行性硬化. 中华肾脏病杂志 (1997)

58. 琨杨, 刘佳丽, 李沁芸, 岳燕林, 张和平. 脂蛋白肾病1例报道及文献复习. 医学诊断 (2019) **9**:6.

59. 杨茜, 葛永纯, 章海涛, 曾彩虹, 程震, 刘志红. 非诺贝特治疗脂蛋白肾病尿检完全缓解3例报告及文献复习. *国际泌尿系统杂志 2016年36卷1期 115-118页 ISTIC* (2016)

60. 李正良, 钟春梅. 脂蛋白肾病二例. 临床肾脏病杂志 (2016) **16**:192.

61. 杨乔焕, 高岩, 欧志英. 脂蛋白肾病1例. 广东医学 (2012) **33**:

62. 杨钢, 刘晓玲, 王汉斌, 刘刚. 脂蛋白肾病. 中国医刊 (2011) **46**:91.

63. 陈伟栋, 黄芬芬. 脂蛋白肾病1例. 中国中西医结合肾病杂志 (2010)

64. 杨聚荣, 何娅妮, 林利容, 李开龙, 丁涵露, 刘晓莉, 许文玲. 免疫吸附治疗重症脂蛋白肾病1例报道. 中国血液净化 (2006) **5**:816–817.

65. 陈晶, 何娅妮, 郭桥楠, 张建国, 赵通武, 刘莉. 脂蛋白肾病1例. 重庆医学 (2003) **032**:699.

66. 高远赋, 刘光陵, 夏正坤, 张连丰, 樊忠明, 伏洁, 付元凤. 脂蛋白肾小球病一例. 中华儿科杂志 (2002) **40**:318–319.

67. 陈楠, 张文, 严静茵, 潘晓霞, 郝翠兰, 傅秀兰. 脂蛋白肾病一例. 上海医学 (2001) **24**:637.

68. 韩鸿玲, 张鹏, 林珊, 邱明才. 脂蛋白肾小球病. 天津医药 (2006) **34**:283–285.

69. Song Y, Yang C, Liu L, Wang H. Case Report: A Pediatric Case of Lipoprotein Glomerulopathy in China and Literature Review. *Front Pediatr* (2021) **9**:684814. doi: 10.3389/fped.2021.684814

70. Takasaki S, Matsunaga A, Joh K, Saito T. A case of lipoprotein glomerulopathy with a rare apolipoprotein E isoform combined with neurofibromatosis type I. *CEN case reports* (2018) **7**:127–131. doi: 10.1007/s13730-018-0309-2

71. Kodera H, Mizutani Y, Sugiyama S, Miyata T, Ehara T, Matsunaga A, Saito T. A Case of Lipoprotein Glomerulopathy with apoE Chicago and apoE (Glu3Lys) Treated with Fenofibrate. *Case reports Nephrol Dial* (2017) **7**:112–120. doi: 10.1159/000478902

72. Usui R, Takahashi M, Nitta K, Koike M. Five-year follow-up of a case of lipoprotein glomerulopathy with APOE Kyoto mutation. *CEN case reports* (2016) **5**:148–153. doi: 10.1007/s13730-016-0214-5

73. Takasaki S, Maeda K, Joh K, Yamakage S, Fukase S, Takahashi T, Suzuki M, Matsunaga A, Saito T. Macrophage Infiltration into the Glomeruli in Lipoprotein Glomerulopathy. *Case reports Nephrol Dial* (2015) **5**:204–212. doi: 10.1159/000441715

74. Tokura T, Itano S, Kobayashi S, Kuwabara A, Fujimoto S, Horike H, Satoh M, Komai N, Tomita N, Matsunaga A, et al. A novel mutation ApoE2 Kurashiki (R158P) in a patient with lipoprotein glomerulopathy. *J Atheroscler Thromb* (2011) **18**:536–541. doi: 10.5551/jat.8102

75. Mitani A, Ishigami M, Watase K, Minakata T, Yamamura T. A novel apolipoprotein E mutation, ApoE Osaka (Arg158 Pro), in a dyslipidemic patient with lipoprotein glomerulopathy. *J Atheroscler Thromb* (2011) **18**:531–535. doi: 10.5551/jat.7377

76. Kinomura M, Sugiyama H, Saito T, Matsunaga A, Sada K, Kanzaki M, Takazawa Y, Maeshima Y, Yanai H, Makino H. A novel variant apolipoprotein E Okayama in a patient with lipoprotein glomerulopathy. *Nephrol Dial Transplant* (2008) **23**:751–756. doi: 10.1093/ndt/gfm675

77. Hagiwara M, Yamagata K, Matsunaga T, Arakawa Y, Usui J, Shimizu Y, Aita K, Nagata M, Koyama A, Zhang B, et al. A novel apolipoprotein E mutation, ApoE Tsukuba (Arg 114 Cys), in lipoprotein glomerulopathy. *Nephrol Dial Transplant* (2008) **23**:381–384. doi: 10.1093/ndt/gfm735

78. Sam R, Wu H, Yue L, Mazzone T, Schwartz MM, Arruda JAL, Dunea G, Singh AK. Lipoprotein glomerulopathy: A new apolipoprotein E mutation with enhanced glomerular binding. *Am J Kidney Dis* (2006) **47**:539–548. doi: 10.1053/j.ajkd.2005.12.031

79. Arai T, Yamashita S, Yamane M, Manabe N, Matsuzaki T, Kiriyama K, Kanayama Y, Himeno S, Matsuzawa Y. Disappearance of intraglomerular lipoprotein thrombi and marked improvement of nephrotic syndrome by bezafibrate treatment in a patient with lipoprotein glomerulopathy. *Atherosclerosis* (2003) **169**:293–299. doi: 10.1016/s0021-9150(03)00194-1

80. Maruyama K, Arai H, Ogawa T, Tomizawa S, Morikawa A. Lipoprotein glomerulopathy: a pediatric case report. *Pediatr Nephrol* (1997) **11**:213–214. doi: 10.1007/s004670050264

81. Ando M, Sasaki J, Hua H, Matsunaga A, Uchida K, Jou K, Oikawa S, Saito T, Nihei H. A novel 18-amino acid deletion in apolipoprotein E associated with lipoprotein glomerulopathy. *Kidney Int* (1999) **56**:1317–1323. doi: https://doi.org/10.1046/j.1523-1755.1999.00677.x

82. Konishi K, Saruta T, Kuramochi S, Oikawa S, Saito T, Han H, Matsunaga A, Sasaki J. Association of a novel 3-amino acid deletion mutation of apolipoprotein E (Apo E Tokyo) with lipoprotein glomerulopathy. *Nephron* (1999) **83**:214–218. doi: 10.1159/000045513

83. Komatsu T, Kanatsu K, Ochi H, Kita T, Doi T. Lipoprotein glomerulopathy with a new apolipoprotein E phenotype. *Am J kidney Dis* (1995) **25**:952–953. doi: 10.1016/0272-6386(95)90581-2

84. Amenomori M, Haneda M, Morikawa J, Nishigaki I, Maeda S, Hidaka H, Kikkawa R, Shigeta Y. A case of lipoprotein glomerulopathy successfully treated with probucol. *Nephron* (1994) **67**:109–113. doi: 10.1159/000187897

85. Saito T, Sato H, Oikawa S, Kudo K, Kurihara I, Nakayama K, Abe K, Yoshinaga K, Sakaguchi H. Lipoprotein glomerulopathy. Report of a normolipidemic case and review of the literature. *Am J Nephrol* (1993) **13**:64–68. doi: 10.1159/000168591

86. Koitabashi Y, Ikoma M, Miyahira T, Fujita R, Mio H, Ishida M, Shimizu K, Sakaguchi H. Long-term follow-up of a paediatric case of lipoprotein glomerulopathy. *Pediatr Nephrol* (1990) **4**:122–128. doi: 10.1007/BF00858822

87. Shibata T, Kaneko N, Hara Y, Saito T, Sakaguchi H. A case of lipoprotein glomerulopathy. Light and electron microscopic observations of the glomerulus. *Acta Pathol Jpn* (1990) **40**:448–457. doi: 10.1111/j.1440-1827.1990.tb01586.x

88. Saito T, Sato H, Kudo K, Oikawa S, Shibata T, Hara Y, Yoshinaga K, Sakaguchi H. Lipoprotein glomerulopathy: glomerular lipoprotein thrombi in a patient with hyperlipoproteinemia. *Am J kidney Dis* (1989) **13**:148–153. doi: 10.1016/s0272-6386(89)80134-9

89. 坂口弘. 第 31 回日本腎臓学会総会 ミニワークショップ: Lipoprotein glomerulopathy. 日本腎臓学会誌 (1989) **31**:451–456.

90. 小松武生, 藤原佳典, 槇林弘之郎, 金津和郎. 網膜中心動脈閉塞を合併した, lipoprotein glomerulopathy の 1 例. 日本内科学会雑誌 (1995) **84**:1332–1333.

91. Ogawa T, Arai H, Watanabe T, Kobayashi Y, Morikawa A, Maruyama K, Hattori H, Egashira T. A new variant of apolipoprotein E in a girl with lipoprotein glomerulopathy. *Japanese J Pediatr Nephrol* (1998) **11**:171–175. doi: 10.3165/jjpn.11.171

92. Hashimoto T, Toyota K, Ogino D, Matsunaga A, Hayasaka K. A second pediatric patient with lipoprotein glomerulopathy carrying a heterozygous APOE-Sendai mutation. *Japanese J Pediatr Nephrol* (2011) **24**:218–223. doi: 10.3165/jjpn.24.218

93. 中井継彦, 松田哲久, 高井博正, 前田肇, 高橋貞夫, 笈田耕治, 玉井利孝, 宮保進, 森河浄, 黒田満彦. ネフローゼ症候群を合併した家族性高コレステロール血症の 1 例. 日本内科学会雑誌 (1990) **79**:1079–1080.

94. 中尾俊之, 友成治夫家口慶彦, 宇都宮正範. Lipoprotein glomerulopathyの 一例. *Japanese J Nephrol* (1990) **32**:1284–1337. doi: 10.14842/jpnjnephrol1959.32.1284

95. 小和瀬貴律, 丸山健一新井英夫，富澤滋. 第22回群馬腎疾患研究会抄録. *KITAKANTO Med J* (1995) **45**:379–382. doi: 10.2974/kmj1951.45.379

96. Morris CS, Bois MC, Aust CH, Thomas R, Sethi S, Maleszewski JJ. Intravascular cardiac lipoproteinosis: extrarenal manifestation of lipoprotein glomerulopathy. *Cardiovasc Pathol* (2019) **42**:6–9. doi: 10.1016/j.carpath.2019.04.006

97. Boumendjel R, Papari M, Gonzalez M. A rare case of lipoprotein glomerulopathy in a white man: an emerging entity in Asia, rare in the white population. *Arch Pathol Lab Med* (2010) **134**:279–282. doi: 10.1043/1543-2165-134.2.279

98. Bomback AS, Song H, D’Agati VD, Cohen SD, Neal A, Appel GB, Rovin BH. A new apolipoprotein E mutation, apoE Las Vegas, in a European-American with lipoprotein glomerulopathy. *Nephrol Dial Transplant* (2010) **25**:3442–3446. doi: 10.1093/ndt/gfq389

99. Sethi S. Renal failure with intracapillary thrombi. Lipoprotein glomerulopathy. *Kidney Int* (2008) **73**:1097–1098. doi: 10.1038/ki.2008.13

100. Pêgas KL, Rohde R, Garcia CD, Bittencourt V de B, Keitel E, Poloni JAT, Cambruzzi E. Lipoprotein glomerulopathy: a case report of a rare disease in a Brazilian child. *J Bras Nefrol* (2014) **36**:93–95. doi: 10.5935/0101-2800.20140015

101. da Silveira-Neto JN, de Oliveira Ahn GJ, de Menezes Neves PDM, Baptista VAF, de Almeida Araújo S, Wanderley DC, Watanabe A, Watanabe EH, Murai NM, Bertollo EMG, et al. Lipoprotein glomerulopathy associated with the Osaka/Kurashiki APOE variant: two cases identified in Latin America. *Diagn Pathol* (2021) **16**:65. doi: 10.1186/s13000-021-01119-x

102. Magistroni R, Bertolotti M, Furci L, Fano RA, Leonelli M, Pisciotta L, Pellegrini E, Calabresi L, Bertolini S, Calandra S. Lipoprotein glomerulopathy associated with a mutation in apolipoprotein e. *Clin Med Insights Case Rep* (2013) **6**:189–196. doi: 10.4137/CCRep.S12209

103. Pasquariello A, Pasquariello G, Innocenti M, Minnei F, Funel N, Lorusso P, Barsotti G. Lipoprotein glomerulopathy: first report of 2 not consanguineous Italian men from the same town. *J Nephrol* (2011) **24**:381–385. doi: 10.5301/JN.2011.7772

104. Russi G, Furci L, Leonelli M, Magistroni R, Romano N, Rivasi P, Albertazzi A. Lipoprotein glomerulopathy treated with LDL-apheresis (Heparin-induced Extracorporeal Lipoprotein Precipitation system): a case report. *J Med Case Rep* (2009) **3**:9311. doi: 10.1186/1752-1947-3-9311

105. Grcevska L, Polenakovic M, Milovanceva-Popovska M, Petrusevska G. New cases of lipoprotein glomerulopathy? *Nephron* (2000) **86**:557–559. doi: 10.1159/000045872

106. Mourad G, Djamali A, Turc-Baron C, Cristol JP. Lipoprotein glomerulopathy: a new cause of nephrotic syndrome after renal transplantation. *Nephrol Dial Transplant* (1998) **13**:1292–1294. doi: 10.1093/ndt/13.5.1292

107. Meyrier A, Dairou F, Callard P, Mougenot B. Lipoprotein glomerulopathy: first case in a white European. *Nephrol Dial Transplant* (1995) **10**:546–549. doi: 10.1093/ndt/10.4.546

108. Andrews PA, O’Donnell PJ, Dilly SA, Snowden SA, Bewick M. Recurrence of lipoprotein glomerulopathy after renal transplantation. *Nephrol Dial Transplant* (1997) **12**:2442–2444. doi: 10.1093/ndt/12.11.2442

109. Marinaki S, Kalaitzakis E, Kolovou K, Gakiopoulou H, Stylianou K, Papasotiriou M, Boletis IN. A case of lipoprotein glomerulopathy in a Greek Caucasian male. *Int Urol Nephrol* (2022) **54**:969–970. doi: 10.1007/s11255-021-02930-7

110. Kollbrunner L, Hirt-Minkowski P, Sanz J, Bresin E, Neuhaus TJ, Hopfer H, Jehle AW. Case Report: Lipoprotein Glomerulopathy Complicated by Atypical Hemolytic Uremic Syndrome. *Front Med* (2021) **8**:679048. doi: 10.3389/fmed.2021.679048

111. Sipovskii VG, Klemina IK, Zverkov R V, Dobronravov VA, Smirnov A V. [A case of diagnosing lipoprotein glomerulopathy in Russia]. *Arkh Patol* (2016) **78**:52–57. doi: 10.17116/patol201678652-57
